# *JCHAIN*: A Prognostic Marker Based on Pan-Cancer Analysis to Inhibit Breast Cancer Progression

**DOI:** 10.3390/genes16091070

**Published:** 2025-09-11

**Authors:** Jinfeng Zhao, Wanquan Chen, Longpeng Li, Zhibin Zhang, Yaxin Wang

**Affiliations:** Institute of Physical Education and Sport, Shanxi University, Taiyuan 030006, China

**Keywords:** *JCHAIN*, immune microenvironment, prognostic marker, IL2-STAT4 signalling pathway, breast cancer

## Abstract

Background/Objectives: The JCHAIN (immunoglobulin-linked chain) is a multimeric IgA and IgM-linked chain whose involvement in oncogenesis and immunomodulation is unknown. The goal of this work was to conduct a comprehensive pan-cancer analysis of the *JCHAIN* to determine its expression profile, prognostic significance, immune infiltration, and function in diverse malignancies. Methods: We performed pan-cancer analysis of gene expression data and protein expression data of *JCHAIN* using multiple databases, and analysed the prognostic significance of *JCHAIN* in a variety of cancers using univariate Cox analysis and Kaplan–Meier tools. The relationship between *JCHAIN* and immune cell infiltration was analysed via the TISIDB and TIMER websites, while single-cell and spatial transcriptomic analyses were performed to analyse the relationship between *JCHAIN* and the immune microenvironment. Mutations in the *JCHAIN* and their connection with methylation were then investigated using the cBioPortal and UALCAN websites. Afterwards, the function of *JCHAIN* was analysed by KEGG as well as GSEA, and the function of *JCHAIN* in breast cancer cells was verified by in vitro experiments. Results: The expression of the *JCHAIN* gene shows significant differences in most cancers, and its high expression is associated with a favourable prognosis. In most cancers, *JCHAIN* gene expression is closely linked to immune-related genes, immune cells, and methylation, as well as to being affected by mutations. In breast cancer, we found that the *JCHAIN* was negatively correlated with cellular stemness. Enrichment analysis indicated that the *JCHAIN* was involved in immune responses, B cell activation, and JAK-STAT signalling pathways. Functional experiments showed that overexpression of the *JCHAIN* inhibited tumour migration and invasion, which may be closely related to the activation of the IL-2/STAT4 signalling pathway. Conclusions: We found that *JCHAIN* can be used as a diagnostic and prognostic marker for a variety of cancers by pan-cancer analysis and verified that *JCHAIN* affects breast cancer cell progression through IL-2/STAT4 by in vitro experiments.

## 1. Introduction

According to the latest estimates of the International Agency for Research on Cancer (IARC), there were nearly 20 million new cancer cases and 9.7 million cancer deaths in 2022 [1]. Currently, significant progress has been made in the treatment of cancer with surgery, targeted therapy, and chemotherapy [2], However, cytotoxicity and drug resistance can occur during treatment [3], and the degree of recurrence and metastasis after treatment varies greatly by cancer stage, which seriously affects the prognosis of patients [4]. Therefore, the identification of novel diagnostic and prognostic biomarkers is important for improving the safety and efficacy of clinical treatments.

The *JCHAIN* gene is located on the long arm of chromosome 4 and is transcriptionally translated into the JCHAIN protein. Previous research has demonstrated that JCHAIN proteins can bind to areas of Immunoglobulin A (IgA) or Immunoglobulin M (IgM) crystallisable fragments, forming IgA dimers or IgM pentamers to promote mucosal immunity [5]. It is worth noting that the JCHAIN protein is expressed not only during the differentiation and development of immunoglobulin-secreting cells (e.g., lymphocytes), but also in non-immunoglobulin-secreting cells such as mammary epithelial cells, dendritic cells, and so on [6]. These findings show that *JCHAIN* may play a crucial function in a range of malignancies and immune cells. A plasma proteomics study revealed that JCHAIN could be exploited as a possible biomarker for multiple myeloma [7]. It can also be employed as a diagnostic and immunoprognostic marker in lung adenocarcinoma (LUAD) [8], ovarian cancer (OV) [9], and cholangiocarcinoma (CHOL) [10] when combined with other genes in prognostic models. Furthermore, *JCHAIN* inhibits breast cancer (BRCA) growth and metastasis via the NF-KB signalling pathway [6].

Given that the above studies were tightly confined to a single cancer, our study explored the role of the *JCHAIN* in multiple cancers. We used the Cancer Genome Atlas (TCGA), Genotype-Tissue Expression (GTEx), and Clinical Proteomics Tumour Analysis Consortium (CPTAC) databases to analyse *JCHAIN* gene expression, protein expression, and prognostic significance. We also examined the relationship between *JCHAIN* and gene mutations, methylation analysis, and immune infiltration across various cancer types, and employed single-cell and spatial transcriptomics to investigate the association between *JCHAIN* and the immune microenvironment in BRCA. Subsequently, we conducted enrichment analyses of *JCHAIN* using Kyoto Encyclopedia of Genes and Genomes (KEGG) and Gene Set Enrichment Analysis (GSEA) and validated the mechanism by which *JCHAIN* inhibits breast cancer through in vitro experiments.

## 2. Materials and Methods

### 2.1. Data Acquisition and Processing

Gene expression data for 33 malignancies, as well as clinical data, were gathered from The Cancer Genome Atlas (TCGA) database (https://portal.gdc.cancer.gov/, accessed on 10 June 2024), GSE10780, GSE109169, GSE134359, GSE29044, GSE50428, GSE53752, GSE54002, GSE61304, GSE65194, GSE76250, GSE139038 and GSE45827 data downloaded from GEO database (https://www.ncbi.nlm.nih.gov/geo/, accessed on 16 June 2024) and analysed using R 4.3.0.

### 2.2. Gene Expression Analysis

We analysed the *JCHAIN* gene expression data in 33 cancers through the Spaekle (https://grswsci.top/, accessed on 10 June 2025) [11]. Additionally, the UALCAN tool (https://ualcan.path.uab.edu/index.html, accessed on 16 May 2025) [12] was used to measure the expression of the JCHAIN protein in nine malignancies. A comprehensive database that offers comprehensive details on the expression and location of human proteins is the Human Protein Atlas (HPA, https://www.proteinatlas.org/, accessed on 16 May 2025) [13]. The “Tissue” and “Cancer” modules in HPA were used to download immunohistochemistry images of the *JCHAIN* for this investigation. Through its “Stubtype” module, Tumor Immune System Interaction Database (TISIDB, http://cis.hku.hk/TISIDB/index.php, accessed on 16 May 2025) [14], a portal for tumour-immune system interactions, thoroughly examines the *JCHAIN* expression trends in immunological and molecular subtypes. The immune subtypes include C1 (wound healing), C2 (IFN-gamma dominant), C3 (inflammatory), C4 (lymphocyte depleted), C5 (immunologically quiet), C6 (TGF-b dominant).

### 2.3. Survival Prognosis Analysis

The prognostic relevance of the *JCHAIN* expression as a predictor of overall survival (OS), disease-specific survival (DSS), progression-free interval (PFI), and disease-free interval (DFI) was assessed in this study using one-way Cox regression analysis. The survival package (version 3.6.4) for the R language (version 4.4.1) carried out the Kaplan–Meier survival analysis method, and the median of *JCHAIN* expression was used to separate the patients into a high and a low expression group in order to assess the survival differences between the two groups. Log-rank tests were used to assess whether the difference in survival curves between the two groups was statistically significant. The pROC package in R was utilised to conduct the ROC analysis. The TCGA-BRCA cohort, GSE139038, and GSE45827 were the three datasets used to analyse the diagnostic utility of the *JCHAIN* in BRCA using ROC curves. We constructed *JCHAIN*-related column Nomogram and calibration curves in breast cancer. KM-Plotter (https://kmplot.com/analysis/, accessed on 18 May 2025) [15] is a web-based application for survival analysis in cancer research. We used this technique to assess the survival of the *JCHAIN* in BRCA on three datasets: GSE1456, GSE20685, and GSE58812.

### 2.4. Analysis of Genetic Modifications

In this study, “TCGA pan-cancer mapping study” was chosen from the “Quick Selection” option on the cBioPortal website (https://www.cbioportal.org/, accessed on 19 May 2025) [16], After that, enter *JCHAIN* into the “Query” module to obtain the mutation landscape, mutation type, and prognosis for *JCHAIN*. Following that, the Sparkle database was used to calculate the correlation of the *JCHAIN* expression with scores such as aneuploidy and homologous recombination defects, and the fmsb package generated a spider web graph to illustrate the pan-cancer correlation coefficients [17]. In addition, we collected 30 homologous recombination repair (HRR)-related genes [18] (Table S1) and analysed the correlation between these genes and the expression of the *JCHAIN* genes by the GEPIA2 tool [19].

### 2.5. Methylation Analysis of JCHAIN in Pan-Cancer

The UALCAN tool was used to assess the JCHAIN promoter methylation in 15 malignancies. *JCHAIN* was also examined using a heatmap in the “Gene-Correlation” module of the TIMER3 website (https://compbio.cn/timer3/, accessed on 25 May 2025) [20] in connection to 44 N1-methyladenosine (m1A), 5-methylcytosine (m5C)and N6-methyladenosine (m6A) modifier gene expression [21] (Table S2).

### 2.6. Correlation of JCHAIN with Cell Stemness in BRCA

By obtaining data on the cellular stemness index of BRCA in a previous article [22], Including mRNA expression-based stemness index (mRNAsi), DNA methylation-based stemness index (mDNAsi) (Differential Methylation Probe Stemming Index (DMPsi), Enhancer Stemming Index (ENHsi), Epigenetically Regulated mDNAsi (EREG-mDNAsi). and using the median of the cellular stemness index as the cutoff value, the samples were divided into high-score and low-score groups. The association of the *JCHAIN* with these data was then investigated.

### 2.7. Immunoinfiltration Analysis of JCHAIN

The Sparkle database was used to first examine the correlation of the *JCHAIN* with chemokine, chemokine receptor, immunoinhibitor, immunostimulator, and major histocompatibility complex (MHC) in each of the designated cancers. Using the ComplexHeatmap package for heat mapping. A heat map of the correlation between *JCHAIN* gene expression and lymphocytes was later obtained in the TISIDB website. Since CD8+ T cells, B cells, macrophages, and Treg cells showed the strongest correlation with *JCHAIN*, these cells were evaluated using heat maps from the TIMER3 website for their association with *JCHAIN* as well as stroma, immune, and estimate scores with the *JCHAIN*. The UALCAN programme was then used to create box plots of the JCHAIN protein expression and immune-related pathways. The TISIDB website provided the correlation scatter plots of the *JCHAIN* with CCL19, CCR2, BTLA, TNFRSF17, and HLA-DOB. The CIBERSORT, ssGSEA, and ESTIMATE algorithms were used to assess the correlation of the JCHAIN with immune cells and immunological scores in BRCA.

### 2.8. Single-Cell Analysis of JCHAIN and Spatial Transcriptome Analyses

To evaluate the *JCHAIN* expression in various cell types, the single-cell database TISCH2 (http://tisch.comp-genomics.org/, accessed on 10 June 2025) was used. Additionally, the Sparkle database (https://grswsci.top/analyze, accessed on 14 June 2025) was used to investigate the spatial distribution of *JCHAIN*.

### 2.9. Enrichment Analysis of JCHAIN

The GO and KEGG enrichment analyses were performed by splitting the median *JCHAIN* expression in TCGA-BRCA into high and low expression groups and then filtering out the differential genes (log2FoldChange > 1, *p* < 0.05) through the clusterProfiler package(Version4.14.3) and the org.Hs.eg.db package(Version3.19.1) [23]. The correlation with *JCHAIN* expression was investigated by obtaining 658 pathways from the KEGG MEDICUS website, enriching the top 12 activated and inhibited pathways using the aforementioned R package, and computing the scores of 12 pathways using the “GSVA” package (Version1.48.0) [24]. The STRING database (https://string-db.org/, accessed on 3 June 2025) [25] was used to find proteins that interact with *JCHAIN*.

### 2.10. Cell Transfection

The *JCHAIN* plasmid was produced (Shanghai Abbott Biotechnology Co., Ltd., Shanghai, China), and cells were transfected using the TransLntro^®^ EL Transfection Kit (TrnasGen Biotech, Beijing, China) according to the instructions, followed by protein measurement.

### 2.11. CCK-8 Experiment

The MCF-7 cells with more than 70% apposition rate, trypsin digestion, centrifugation at 1000 rpm for 5 min, take the precipitate and dilute the concentration to 1000–2000 cells per 1 mL, take a 96-well plate, add 200 μL of cell suspension to each well, incubate in the incubator for 24 h, and add l0 μL of CCK-8 in each well at 24 h, 48 h, and 72 h, respectively. In each well, add a solution (BMU106-CN, Abbkine, Wuhan, China) and incubate for 4 h before measuring absorbance at 450 nm using an enzyme marker (30050303, TECAN, Männedorf, Switzerland).

### 2.12. Cell Scratching Assay

Cells were inoculated into 24-well plates, and when the wall-adherence rate of adherent cells reached 70–80%, the cells were scored by scratching experiments using a 200 μL tip and scratched 3 times, and photographed with an inverted fluorescence microscope at 0 h. Subsequently, the cells were cultured in an incubator for 24 h and 48 h, and observed and photographed, respectively, and the cell mobility was calculated by ImageJ (1.53e, Bethesda, MD, USA) using a scratch width Calculation formula: (0 h width − 24 h width)/0 h width × 100%.

### 2.13. Experiments in Colony Generation

Cells were inoculated into 6-well plates (4000/well) and cultured for 1–2 weeks. After colony generation, supernatant was removed, 1 mL of 4% paraformaldehyde was added to each well for 15 min of fixation, paraformaldehyde was removed, 1 mL of crystal violet solution was added to each well for 15 min of staining, the cells were washed with PBS until the purple colour did not appear on the PBS, air dried, and photographed.

### 2.14. Western Blot

MCF-7 breast cancer cells were lysed with RIPA buffer, and then the total protein concentration was determined by BCA protein assay kit (Shanghai, China). Equal amounts of proteins were separated by SDS-polyacrylamide gel electrophoresis (SDS-PAGE) and transferred to a PVDF membrane (Millipore, Billerica, MA, USA). It was blocked with 5% skimmed milk for 2 h at room temperature. Diluted primary antibodies were then added and overnight at 4 °C. The membrane was washed and incubated with the corresponding peroxidase (HRP-conjugated) secondary antibody (1:5000) for 1 h. The signals were detected with an ECL detection kit (Applygen Technologies Inc, Beijing, China) and imaged in a chemiluminescence metre (ChemiDoc XRS+, Bio-Rad, Hercules, CA, USA). β- actin was used as an endogenous control. The following primary antibodies were used: STAT4 (1:1000, item number A4523), p-JAK3 (1:1000, item number AF8160), JAK3 (1:2000, item number AF0008), JCHAIN (1:1000, item number ab269855), IL-2 (1:5000, item number 83558-5RR), β- actin (1:100,000, stock number 81115-1-RR), where β-actin and IL-2 were from Proteintech Group, Inc. (Wuhan, China), STAT4 and JCHAIN were from ABclonal Technology Co., Ltd. (Wuhan, China) and abcam (Shanghai, China), respectively, and p-JAK3 and JAK3 from Affinity Biosciences (Jiangsu, China).

### 2.15. Transwell Test

Transwell chambers (BIOFIL) were used for the Transwell assay. The matrix gel was spread on the upper layer of the Transwell chambers by diluting the matrix gel with serum-free medium (100 uL of matrix gel was added to 800 uL of medium), mixing, and solidifying in the incubator for 2–4 h. The culture was changed to blood-free medium one day in advance and the cells were starved for 12 h. Afterwards, the cells were detached from the dishes with trypsin and the cell suspension (2.5 × 10^5^ cells/mL) was configured by adding 100 µL of cell suspension to the upper chamber of the Transwell and 500 µL of medium containing 10% FBS to the lower chamber. The plates were placed in an incubator at 37 °C with 5% CO_2_ for 48 h. Cells were fixed with 4% paraformaldehyde, stained with crystal violet, and observed under a microscope and counted.

### 2.16. Protein-Protein Molecular Docking

The 3D structures of JCHAIN (P01591) and IL-2 (P05016) were acquired by downloading them from the AlphaFold Protein Structure Database (https://alphafold.ebi.ac.uk/, accessed on 25 May 2025) [26] and using the GRAMM website (https://gramm.compbio.ku.edu/, accessed on 25 May 2025) [27] after protein-protein molecule docking. PYMOL software (Version 2.6.0a0) was then used to display amino acid residues up to 5A.

### 2.17. RT-PCR

Tissue samples from breast cancer patients were provided by Shanxi Cancer Hospital, and the study was approved by the Research Ethics Committee of Shanxi Cancer Hospital (approval number: KY2023163). All participants signed an informed consent form. Total RNA was extracted from the tissues by using the TransZol UP kit (ET111, TrnasGen Biotech, Beijing, China). cDNA synthesis was performed using the TransScript^®^ Uni All-in-One First-Strand cDNA Synthesis SuperMix for qPCR kit (AU341, TrnasGen Biotech) for cDNA synthesis at 42–65 °C. This was followed by PCR assay by PerfectStart^®^ Green qPCR SuperMix kit (AQ601, TrnasGen Biotech), after which measurements were made by using a fluorescent quantitative PCR instrument (QuantStudio, Thermo Fisher Scientific, Shanghai, China), after which the results were analysed using the 2-ΔΔCq method for normalisation. The primer sequences were JCHAIN: 5′-ACCATTTGCTTTTCTGGGG AGTCC-3′ and 5′-CGGAAGAACGGATGATGATCCTG GAAG-3′. GAPDH. 5′-TCAAC GACCACTTTGTCAAGCTCA-3′ and 5′-GCTGGTGGTCC AGGGGGTCTTACT-3′.

### 2.18. Statistical Analysis

Comparisons between two groups were made using Student’s *t*-test for data that conformed to a normal distribution, and Wilcoxon’s rank sum test (for independent samples) for non-normally distributed data. Comparisons between multiple groups were made using the Kruskal–Wallis test. For all high-throughput analyses involving Multiple Comparisons hypothesis testing (differential gene expression analyses), *p*-values were corrected using the Benjamini–Hochberg (BH) method to control for false discovery rate (FDR). These analyses used FDR-corrected *p*-values (q-values) < 0.05 as the criterion for statistical significance. For all other pre-determined individual comparisons, a two-sided *p*-value < 0.05 was used as the criterion for significance. All statistics were statistically analysed using R 4.3.0.

## 3. Results

### 3.1. Gene Expression of the JCHAIN in Pan-Cancer

**Figure 1:**
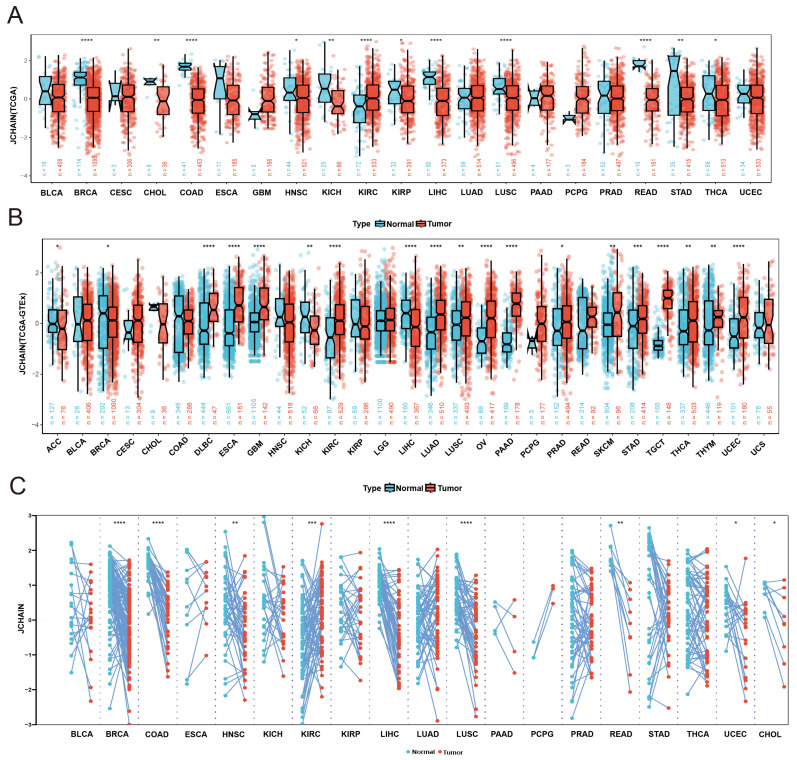
*JCHAIN* mRNA expression in pan-cancer. (**A**) *JCHAIN* expression in 21 cancers in the TCGA dataset. (**B**) TCGA and GTEx dataset tumour and healthy tissue *JCHAIN* expression analysis. (**C**) *JCHAIN* expression of paired samples in the TCGA dataset. Wilcoxon Rank Sum Tests. * *p* < 0.05, ** *p* < 0.01, *** *p* < 0.001, **** *p* < 0.0001.

**Figure 2:**
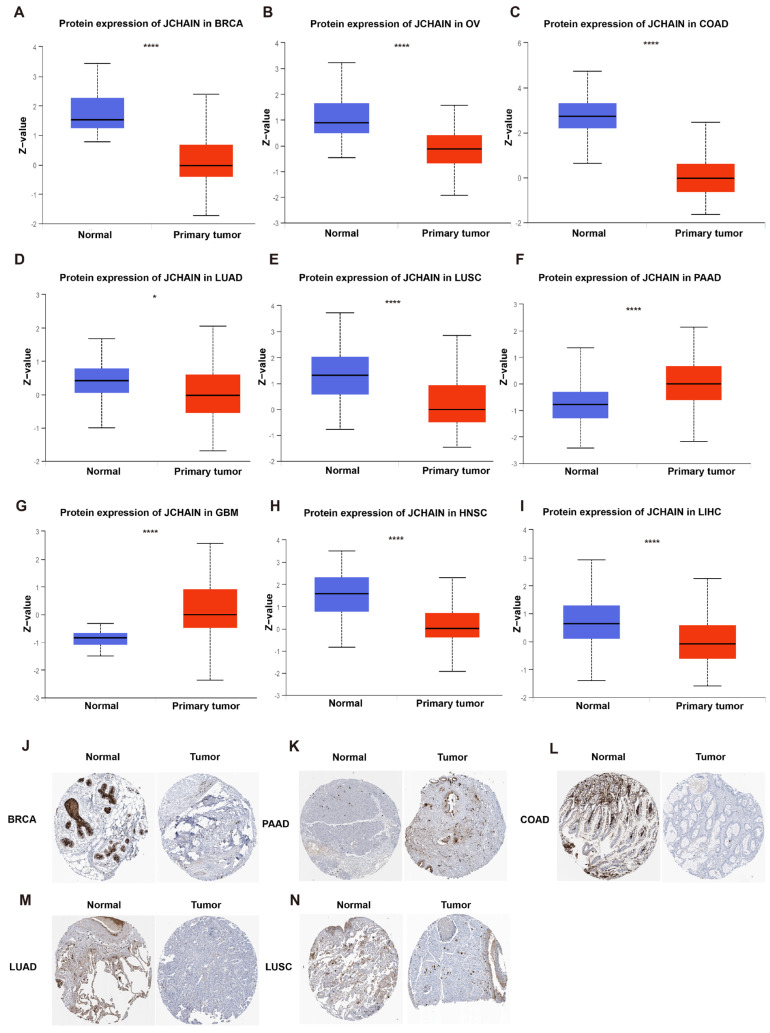
Protein expression of JCHAIN in pan-cancer. (**A**−**I**) In the UALCAN tool, JCHAIN protein expression in tumours including BRCA (**A**), OV (**B**), COAD (**C**), LUAD (**D**), LUSC (**E**), PAAD (**F**), GBM (**G**), HNSC (**H**), and LIHC (**I**). (**J**−**N**) The immunohistochemistry images of JCHAIN in BRCA (**J**), PAAD (**K**), COAD (**L**), LUAD (**M**), and LUSC (**N**) on the HPA website. Student’s *t*-test. * *p* < 0.05, **** *p* < 0.0001.

**Figure 3:**
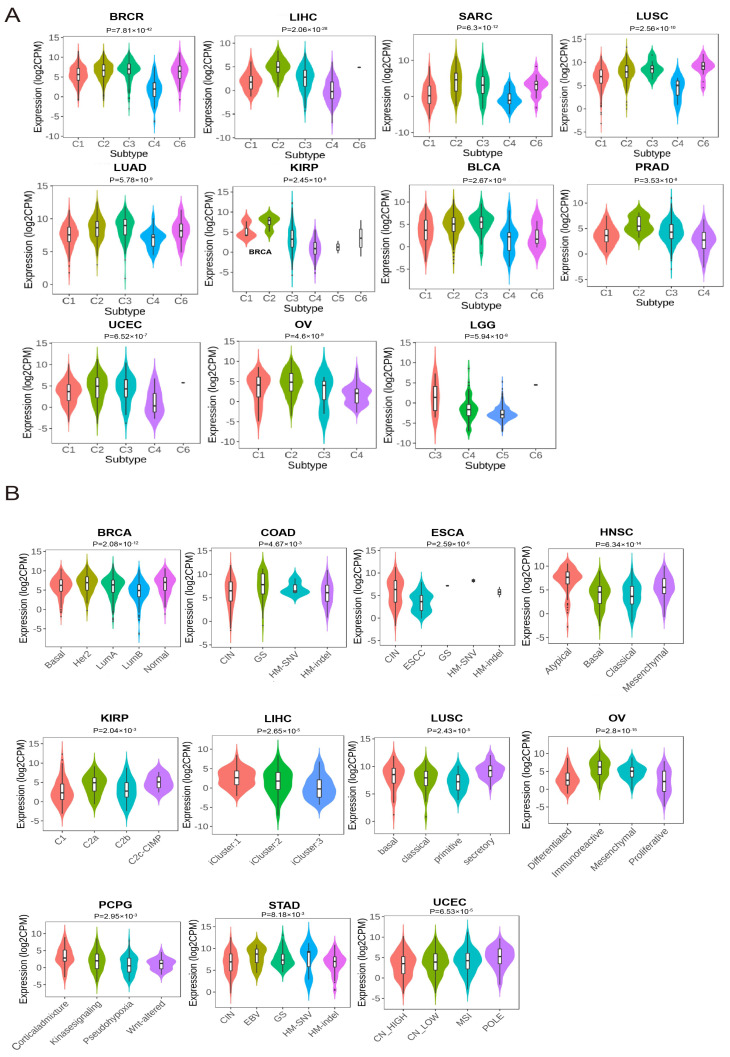
*JCHAIN* expression in immune subtypes and molecular subtypes of pan-cancer. (**A**) Expression of *JCHAIN* in 11 cancers (BRCA, LIHC, SARC, LUSC, LUAD, KIRP, BLCA, PRAD, UCEC, OV, LGG) for immune subtypes was analysed at the TISIDB website. The immune subtypes include C1 (wound healing), C2 (IFN-gamma dominant), C3 (inflammatory), C4 (lymphocyte depleted), C5 (immunologically quiet), C6 (TGF-b dominant). (**B**) Expression of *JCHAIN* for molecular subtypes in 11 cancers (BRCA, COAD, ESCA, HNSC, KIRP, LIHC, LUSC, OV, PCPG, STAD, UCEC) was analysed at the TISIDB website. Kruskal–Wallis test.

To explore the expression profile of the *JCHAIN* in cancer, we analysed *JCHAIN* gene expression data in 33 cancers from the TCGA database, the *JCHAIN* was shown to be lowly expressed in BRCA, CHOL, Colon Adenocarcinoma (COAD), Head and Neck Squamous Cell Carcinoma (HNSC), Kidney Chromophobe (KICH), Kidney Renal Papillary Cell Carcinoma (KIRP), Liver Hepatocellular Carcinoma (LIHC), Lung Squamous Cell Carcinoma (LUSC), Rectum Adenocarcinoma (READ), Stomach Adenocarcinoma (STAD), and Thyroid Carcinoma (THCA), but highly expressed in Kidney Renal Clear Cell Carcinoma (KIRC) (Figure 1A,C). Because some tumours in the TCGA database lacked normal tissues, TCGA was combined with GTEx to investigate the expression of *JCHAIN* in individual cancers, and *JCHAIN* was found to be lowly expressed in Adrenocortical Carcinoma (ACC), BRCA, KICH, LIHC and highly expressed in Diffuse Large B cell Lymphoma (DLBC), Esophageal Carcinoma (ESCA), Glioblastoma Multiforme (GBM), KIRC, Lung Adenocarcinoma (LUAD), LUSC, OV, Pancreatic Adenocarcinoma (PAAD), Prostate Adenocarcinoma (PRAD), Skin Cutaneous Melanoma (SKCM), STAD, Testicular Germ Cell Tumours (TGCT), THCA, Thymoma (THYM), and Uterine Corpus Endometrial Carcinoma (UCEC) (Figure 1B). The expression of JCHAIN protein in several malignancies was subsequently examined using the UALCAN tool and the Human Protein Atlas (HPA), and it was discovered that JCHAIN protein was lowly expressed in BRCA, OV, COAD, LUAD, LUSC, HNSC, LIHC, and highly expressed in PAAD and GBM (Figure 2A–J). These findings indicate that the *JCHAIN* plays a significant role in a range of malignancies. Then, using the “Subtype” module on the TISIDB website, we examined *JCHAIN* expression in relation to immunological and molecular subtypes in each cancer. The immune subtypes were classified as C1 (wound healing), C2 (IFN-gamma dominant), C3 (inflammatory), C4 (lymphocyte deficient), C5 (immunologically silent), and C6 (TGF-b dominant). Our analysis determined the correlation between *JCHAIN* mRNA expression and immunophenotyping, including BRCA, LIHC, SARC, LUSC, OV, LUAD, KIRP, BLCA, PRAD, LGG, and UCEC, and differential phenotypes of *JCHAIN* expression could be found in different immunosubtypes within the same cancer, e.g., in BRCA, C1-C3, C6 expression was significantly higher, while C4 expression was lower (Figure 3A). The molecular subtypes of BRCA, OV, HNSC, ESCA, LIHC, LUSC, UCEC, KIRP, PCPG, COAD, and STAD were significantly correlated with *JCHAIN* expression, implying that *JCHAIN* may play an important role in the tumour microenvironment and influence the molecular subtypes of individual tumours (Figure 3B).

### 3.2. Prognostic Analysis of JCHAIN in Pan-Cancer

**Figure 4:**
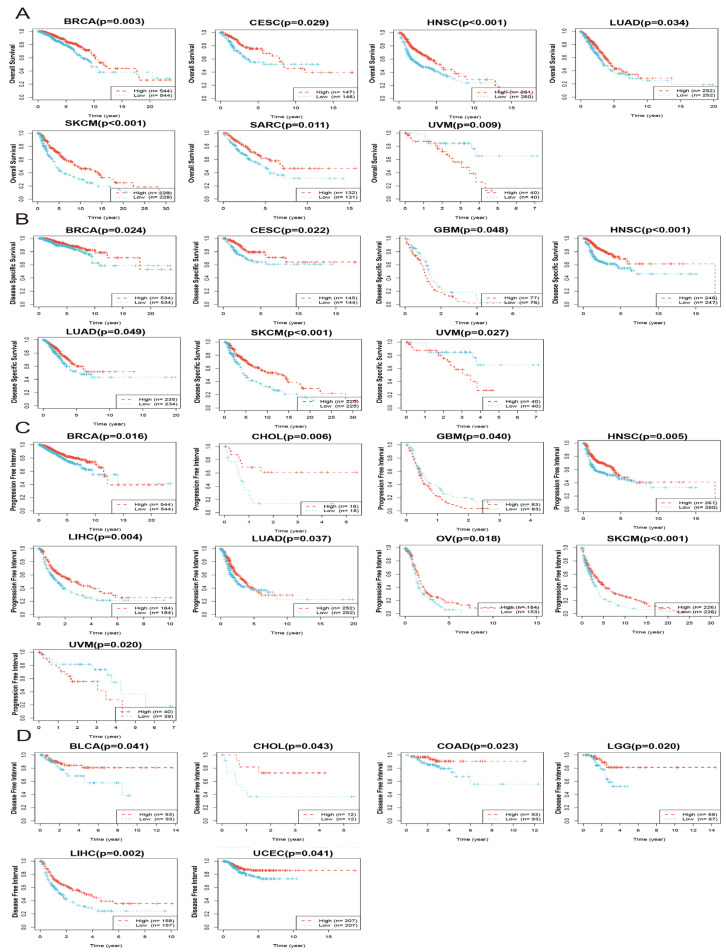
Prognosis of *JCHAIN* in pan-cancer (TCGA). (**A**) The overall survival of *JCHAIN* in 7 cancers including BRCA, CESC, HNSC, LUAD, SKCM, SARC and UVM. (**B**) The disease-specific survival of *JCHAIN* in 7 types of cancers including BRCA, CESC, HNSC, GBM, LUAD, SKCM and UVM. (**C**) The progression free interval of *JCHAIN* in 9 types of cancers including BRCA, CHOL, GBM, HNSC, LIHC, LUAD, OV, SKCM and UVM. (**D**) The disease-free interval of *JCHAIN* in 6 cancers including BLCA, CHOL, COAD, LGG, LIHC and UCEC.

We analysed *JCHAIN* expression and prognosis in various cancers. This included overall survival (OS), disease-specific survival (DSS), progression-free interval (PFI), and disease-free interval (DFI). The findings in Figure 4A indicate that while high expression of *JCHAIN* in UVM was linked to poor overall survival, high expression of *JCHAIN* in BRCA, CESC, HNSC, LUAD, SARC, and SKCM was linked to good overall survival. In disease-specific survival analysis, increased *JCHAIN* expression in BRCA, Cervical Squamous Cell Carcinoma and Endocervical Adenocarcinoma (CESC), HNSC, LUAD, and SKCM resulted in good DSS, whereas the inverse was true in GBM and Uveal Melanoma (UVM) (Figure 4B). In addition, high *JCHAIN* expression in BRCA, CHOL, HNSC, LIHC, LUAD, OV, and SKCM was associated with excellent PFI, whereas the opposite was true in GBM and UVM. The relationship between DFI and *JCHAIN* expression was later investigated, and it was discovered that high *JCHAIN* expression in Bladder Urothelial Carcinoma (BLCA), CHOL, COAD, Brain Lower Grade Glioma (LGG), LIHC, and UCEC was associated with good DFI (Figure 4D). One-way Cox regression scores were also used to analyse the connection between *JCHAIN* and the four prognoses (Figure S1A–D). In conclusion, the *JCHAIN* expression was highly related to prognosis.

### 3.3. The JCHAIN Gene Mutations in Pan-Cancer

**Figure 5:**
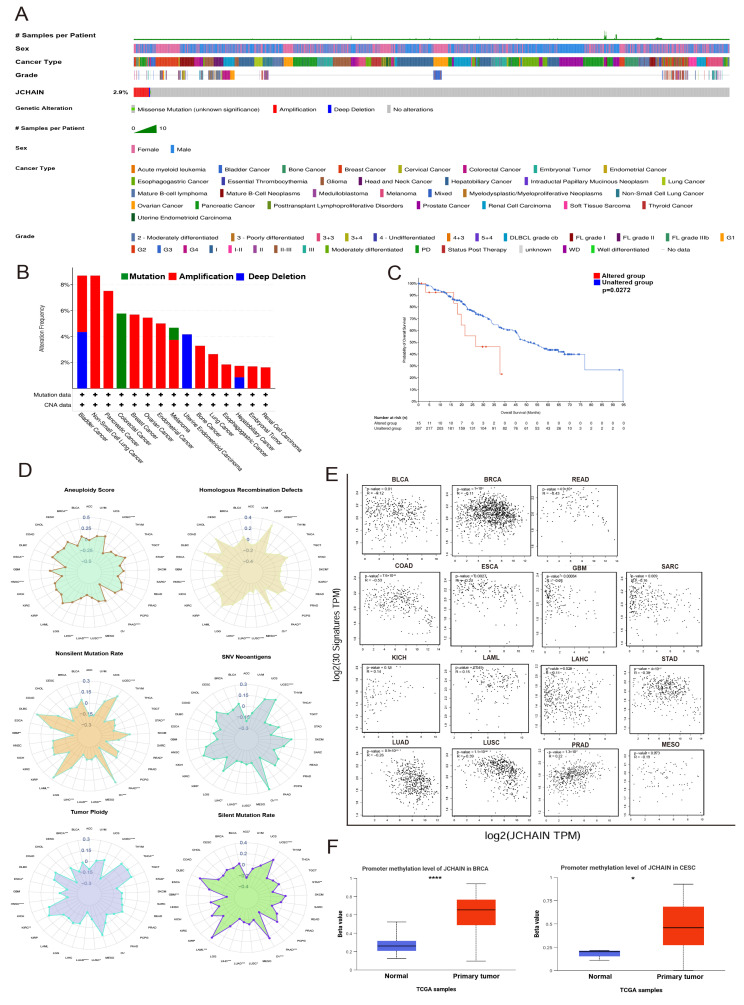
Genetically altered features of *JCHAIN* and methylation. (**A**) Mutation Landscape of *JCHAIN* in cBioportal database. (**B**) Mutation Types of *JCHAIN* in various cancers. (**C**) KM Curve between Altered group and Unaltered group in *JCHAIN*. (**D**) *JCHAIN* in Aneuploidy, Homologous Recombination Defects (HRD), Nonsilent Mutation Rate, Tumour Ploidy, Silent Mutation Rate, and SNV Neoantigens in the correlation pan-cancer radar plot. (**E**) Correlation Scatter Plot of 15 cancers highlighting the correlation between the 30-gene HRR signature and *JCHAIN* levels. (**F**) Methylation analysis of BRCA as well as CESC in the UALCAN tool. Student’s *t*-test, * *p* < 0.05, ** *p* < 0.01, *** *p* < 0.001, **** *p* < 0.0001.

Genetic modification is a form of epigenetics. We investigated TCGA data in the cBioPortal database and discovered that the genetic modifications of *JCHAIN* in various malignancies were mutation, amplification, and deep deletion. The *JCHAIN* amplification occurred in the majority of malignancies, including BLCA, Non-Small Cell Lung Cancer, PAAD, BRCA, OV, ESCA, SKCM, Sarcoma (SARC), Lung Cancer, ESCA, KIRC, LIHC, and TGCT. Mutations occurred only in COAD and SKCM, while profound deletion occurred in BLCA, UCEC, and LIHC (Figure 5A,B). Additionally, the Altered group’s prognosis was poorer than that of the Unaltered group (Figure 5C). Given the prevalence of mutations in tumours and their potential impact on prognosis and treatment outcomes, the *JCHAIN* was also assessed in relation to Aneuploidy, Homologous Recombination Defects (HRD), Nonsilent Mutation Rate, Tumour Ploidy, Silent Mutation Rate and Single Nucleotide Variants (SNV) Neoantigens, and found to be negatively associated with them in the majority of cancers. Recombination Defects (HRD) and *JCHAIN* expression were positively connected in UCS and OV (Figure 5D). Likewise, it showed a positive correlation with SNV Neoantigens in OV and UCEC, as well as with Nonsilent Mutation Rate and Silent Mutation Rate in LAML, OV, and UCEC (Figure 5D). The correlation between *JCHAIN* and 30 MRR signature-associated genes was then assessed using the GEPIA2 website, and it was discovered that JCHAIN expression was negatively correlated with MRR signature-associated genes in the majority of cancers, and positively correlated only in KICH, LAML, and LAHC (Figure 5E). These findings indicate that *JCHAIN* is significantly associated with genomic instability.

### 3.4. Methylation Analysis of the JCHAIN in Pan-Cancer

To understand its transcriptional regulation in these cancers, we thoroughly examined two regulatory mechanisms: promoter DNA methylation and RNA methylation alterations including N1-methyladenosine (m1A), 5-methylcytosine (m5C), and N6-methyladenosine (m6A). Analysis of promoter DNA methylation performed through the UALCAN site revealed that, compared to normal tissues, the *JCHAIN* promoter methylation was decreased in BLCA, COAD, ESCA, GBM, HNSC, KIRC, LIHC, READ, and THCA, whereas it was increased in BRCA, CESC, LUAD, LUSC, PRAD, and UCEC (Figure 5F and Figure S2A). The *JCHAIN* was then correlated with genes with RNA methylation changes using TIMER 3.0 (Figure S3A–C). The *JCHAIN* was found to be negatively correlated with RNA methylation modification genes in most cancers. These findings indicate that *JCHAIN* plays a key role in DNA and RNA methylation modification in a variety of malignancies.

### 3.5. Immunoinfiltration Analysis of the JCHAIN in Pan-Cancer

**Figure 6:**
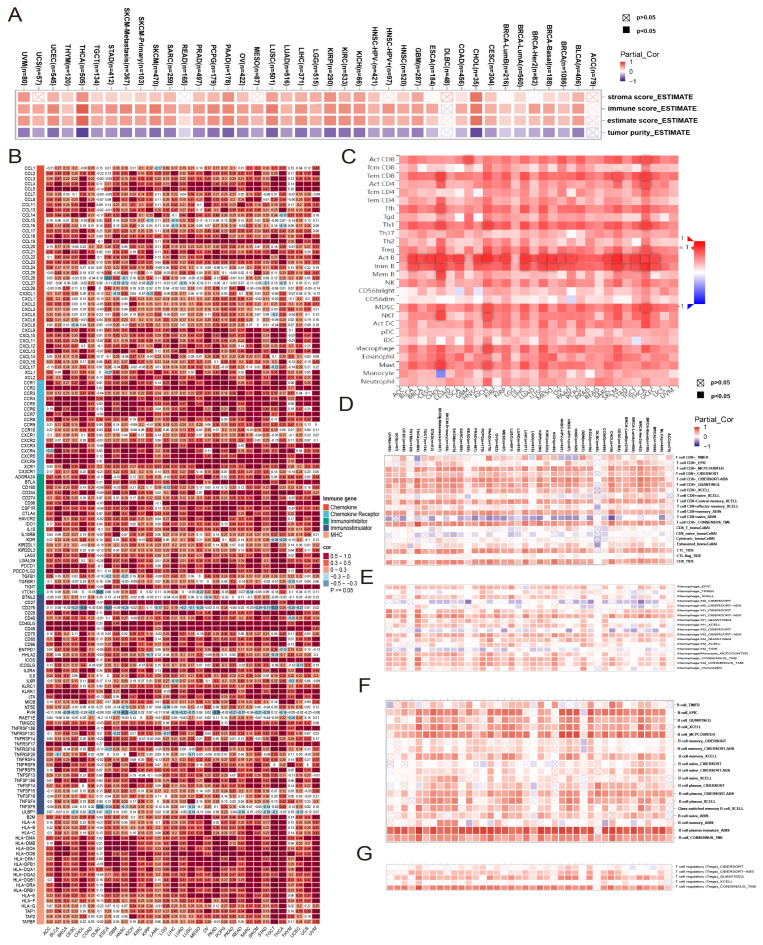
*JCHAIN* immune cell infiltration in pan-cancer. (**A**) Heatmaps of *JCHAIN*’s correlation with stroma, immune, and estimate scores for 33 cancers were analysed in the TIMER3 website. (**B**) Heatmap of *JCHAIN* correlation with Chemokine, Chemokine Receptor, Immunoinhibitor, Immunostimulator, and MHC signature genes in pan-cancer. (**C**) Heatmap of *JCHAIN* correlation with lymphocytes in TISIDB website. (**D**–**G**) Heatmap of *JCHAIN* correlation with CD8+ T-cells, Macrophages, B cells, and Tregs cells in pan-cancer.

To further investigate the relationship between *JCHAIN* and immunity, the relationship between *JCHAIN* and the stroma, immune, and estimate scores of 33 cancers was evaluated using the TIMER 3.0 website, and it was discovered that the three scores were positively correlated in the majority of cancers, but there was no correlation in the ACC or the DLBC (Figure 6A). The correlation between *JCHAIN* and immune-related genes was then investigated further, and it was discovered that *JCHAIN* was positively correlated with the majority of the genes characterised by Chemokine, Chemokine Receptor, Immunoinhibitor, Immunostimulator, and MHC, with the exception of DLBC (Figure 6B). The relationship between *JCHAIN* and immune cells was then investigated utilising the TISIDB’s “Lymphocyte” module and the TIMER3 website. The *JCHAIN* was shown to be positively connected with 28 lymphocytes in 30 cancer types (Figure 6C), and *JCHAIN* was found to be positively correlated with CD8+ T-cells, macrophages, B cells, and Tregs by various algorithms on the TIMER3 website (Figure 6D–G). The JCHAIN proteins were also linked to several immune-related pathways in a range of malignancies, particularly in BRCA, including the WNT pathway, mTOR route, p53 pathway, RTK pathway, Hippo pathway, and chromatin modifiers (Figure S4A). The results presented above indicate that *JCHAIN* may play a significant function in the immunological microenvironment.

### 3.6. Association of the JCHAIN with Pathological Features and Prognosis in BRCA

**Figure 7:**
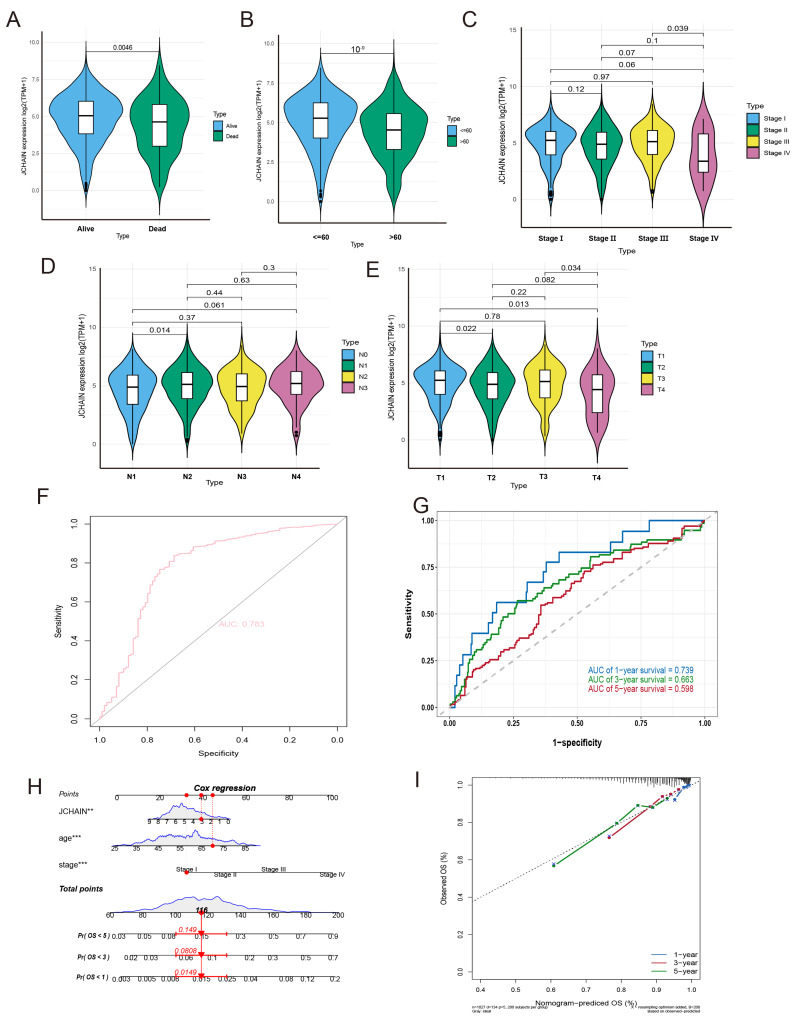
*JCHAIN* in BRCA in relation to pathological features and prognosis. (**A**–**E**) *JCHAIN* expression violin plots for survival status, age, stage, N stage, and T stage. (**F**) *JCHAIN*’s ROC curve. (**G**) *JCHAIN* AUC curves for predicted survival at 1, 3, and 5 years. (**H**) Nomogram of *JCHAIN* constructed with selected clinical features. (**I**) Calibration curves for *JCHAIN*. The Kruskal–Wallis test. ** *p* < 0.01, *** *p* < 0.001.

The above results revealed that *JCHAIN* has a better prognosis in BRCA, with differences in expression, degree of immune infiltration, and high correlation with immune-related pathways, so the role of *JCHAIN* in BRCA was further investigated. In BRCA patients, we examined the relationship between *JCHAIN* and age, survival status, stage staging, N staging, and T staging. Firstly, we revalidated the expression of JCHAIN in breast cancer in the GSE10780, GSE109169, GSE134359, GSE29044, GSE50428, GSE53752, GSE54002, GSE61304, GSE65194, and GSE76250 datasets, and found that the expression of *JCHAIN* was lower in tumours compared to normal samples (Figure S5A). We discovered that poor clinical features were linked to low expression of *JCHAIN* (Figure 7A–E). Furthermore, *JCHAIN*’s prognosis was confirmed in three datasets: GSE1456, GSE20685, and GSE58812, and it was discovered that a good prognosis was linked to high *JCHAIN* expression (Figure S6A). The subsequent AUC values in the TCGA-BRCA cohort, GSE139038, and GSE45827 were 0.783, 0.747, and 0.779, respectively, whereas the area under the curve (AUC) of *JCHAIN* at 1, 3, and 5 years was 0.739, 0.663, and 0.598, respectively, suggesting that *JCHAIN* has good diagnostic value in BRCA (Figure 7F,G and Figure S6B). Following that, we integrated *JCHAIN* expression with a variety of clinical characteristics to generate a nomogram and a calibration curve (Figure 7H,I), both of which were capable of accurately predicting BRCA patient survival.

### 3.7. The JCHAIN in BRCA in Relation to Cell Stemness

**Figure 8:**
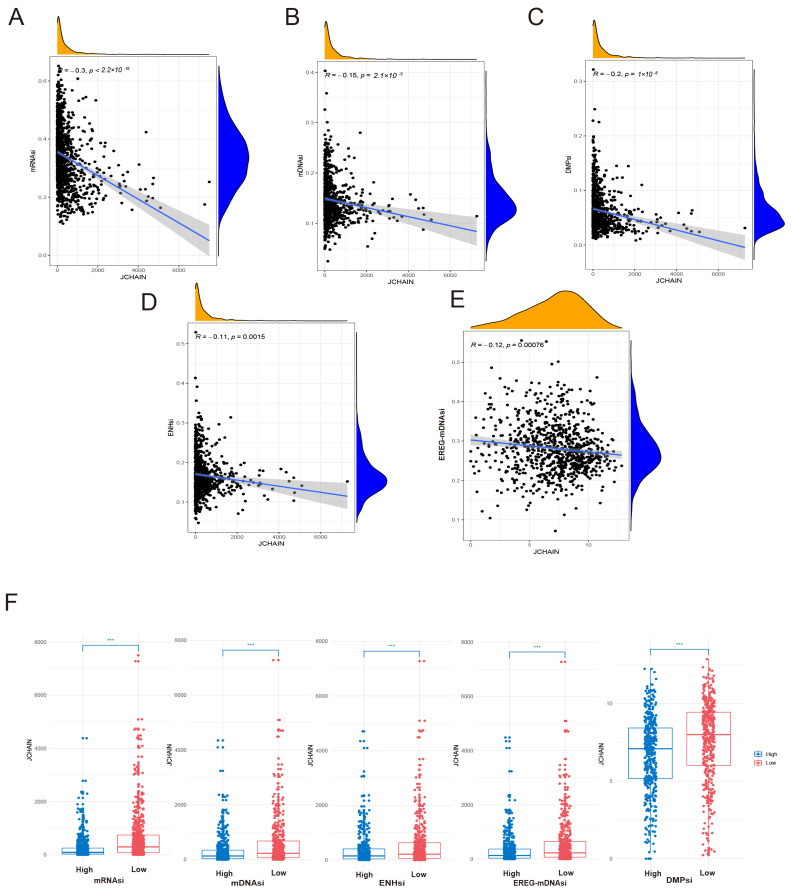
Correlation of *JCHAIN* with cell stemness in BRCA. (**A**–**E**) Scatterplot of correlation between gene expression of *JCHAIN* and mRNAsi, mDNAsi (DNAsi, ENHsi, EREG-mDNAsi) scores in breast cancer. (**F**) mRNAsi and mDNAsi (DNAsi, ENHsi, EREG-mDNAsi) were categorised into high-score and low-score groups, respectively. Box plots showing differences in *JCHAIN* gene expression between the high-score group (blue) and low-score group (red). Wilcoxon Rank Sum Tests. *** *p* < 0.001.

By downloading the cell stemness data corresponding to the TCGA dataset in the relevant literature, we analysed the relationship between *JCHAIN* and cell stemness indices in BRCA and found that *JCHAIN* was negatively correlated with mRNAsi and mDNAsi (DNAsi, ENHsi, and EREG-mDNAsi), which indicated that the low expression of *JCHAIN* promoted breast cancer cell stemness development (Figure 8A–F).

### 3.8. Correlation of the JCHAIN with Immune Cell Infiltration in BRCA

**Figure 9:**
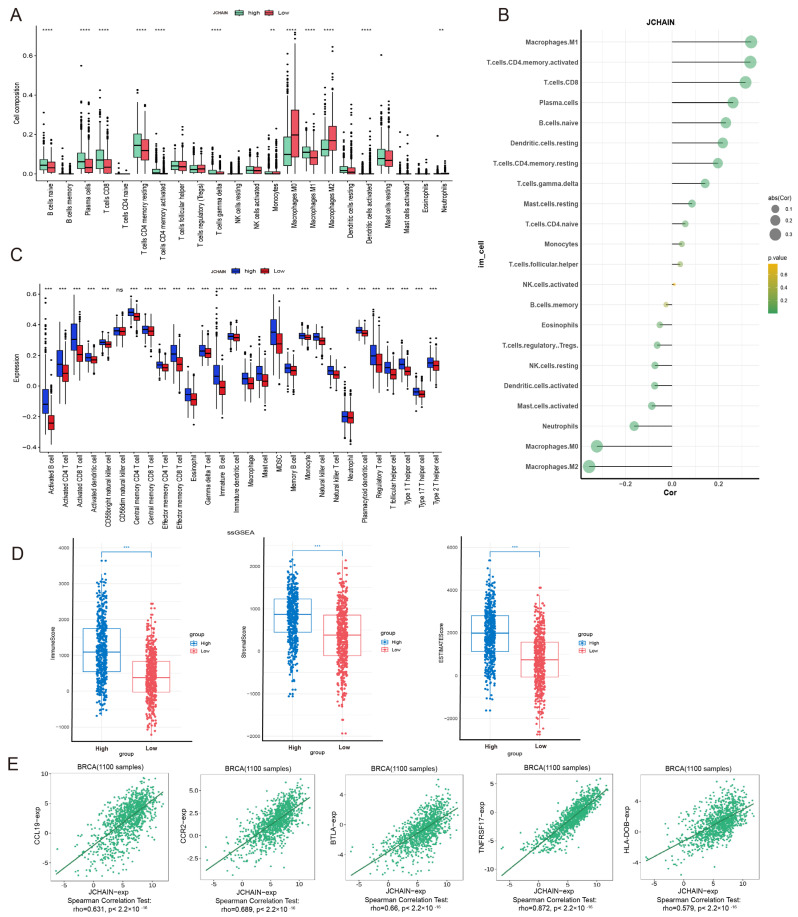
Correlation of *JCHAIN* with immune cell infiltration in BRCA. (**A**) Box plots of 22 immune cells in relation to *JCHAIN* were analysed using the CIBERSORT algorithm. (**B**) Bar graph of *JCHAIN* correlation with 22 immune cells. (**C**) Box plots of 28 immune cells against *JCHAIN* were analysed using the SSCSVA algorithm. (**D**) Box plots of *JCHAIN* in relation to Immune, Stromal and estimate score were analysed using the ESTIMATE algorithm. Wilcoxon Rank Sum Tests. (**E**) Scatterplot of *JCHAIN* in BRCA associated with CCL19, CCR2, BTLA, TNFRSF17, and HLA-DOB. Ns denotes no statistically significant difference. * *p* < 0.05, ** *p* < 0.01, *** *p* < 0.001, **** *p* < 0.0001.

**Figure 10:**
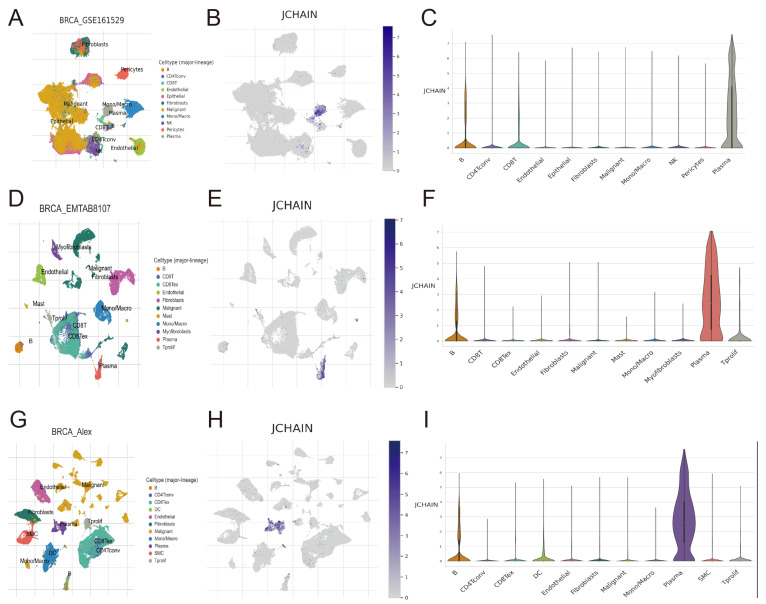
Single-cell analysis of *JCHAIN* in BRCA. (**A**–**C**) Distribution of *JCHAIN* gene expression across different cell types within the BRCA_GSE161529 single-cell dataset (**A**), distribution of *JCHAIN* gene expression (**B**), and expression of *JCHAIN* in different cells (**C**). (**D**–**F**) Distribution of *JCHAIN* gene expression across different cell types within the BRCA_EMTAB8107 single-cell dataset (**D**), distribution of *JCHAIN* gene expression (**E**), and expression of *JCHAIN* in different cells (**F**). (**G**–**I**) Distribution of *JCHAIN* gene expression across different cell types within the BRCA_Alex single-cell dataset (**G**), distribution of *JCHAIN* gene expression (**H**), and expression of *JCHAIN* in different cells (**I**).

Three algorithms—CIBERSORT, ssGSVA, and ESTIMATE—were used to assess the link between immune cells and *JCHAIN*. The results showed that *JCHAIN* was positively correlated with B cells, CD8+ T cells, CD4+ T memory cells, M1 macrophages, and plasma cells. In other words, the low expression group’s immune cell content was lower than that of the high expression group of *JCHAIN*, whereas M0 and M2 macrophages showed a negative correlation with *JCHAIN* and a positive correlation with M1, suggesting that *JCHAIN* was closely linked to macrophage differentiation (Figure 9A–C). The association between *JCHAIN* and stroma, immune, and ESTIMATE scores was then evaluated. It was discovered that the low-scoring group had lower *JCHAIN* expression than the high-expression group of the three scores, indicating that high ESTIMATE scores are linked to high *JCHAIN* expression (Figure 9D). The TISIDB website revealed that in BRCA, the most relevant Chemokine, Chemokine Receptor, Immunoinhibitor, Immunostimulator, and MHC signature genes for *JCHAIN* were CCL19, CCR2, BTLA, TNFRSF17, and HLA-DOB, all of which were positively associated (Figure 9E). Following that, the intracellular expression of *JCHAIN* was examined using single-cell analysis, and it was discovered that *JCHAIN* expression was highest in B cells and plasma cells and lowest in other immune cells (Figure 10A–I). The results showed that increased *JCHAIN* expression was related with a healthy immunological microenvironment.

### 3.9. Spatial Transcriptomics in the JCHAIN

**Figure 11:**
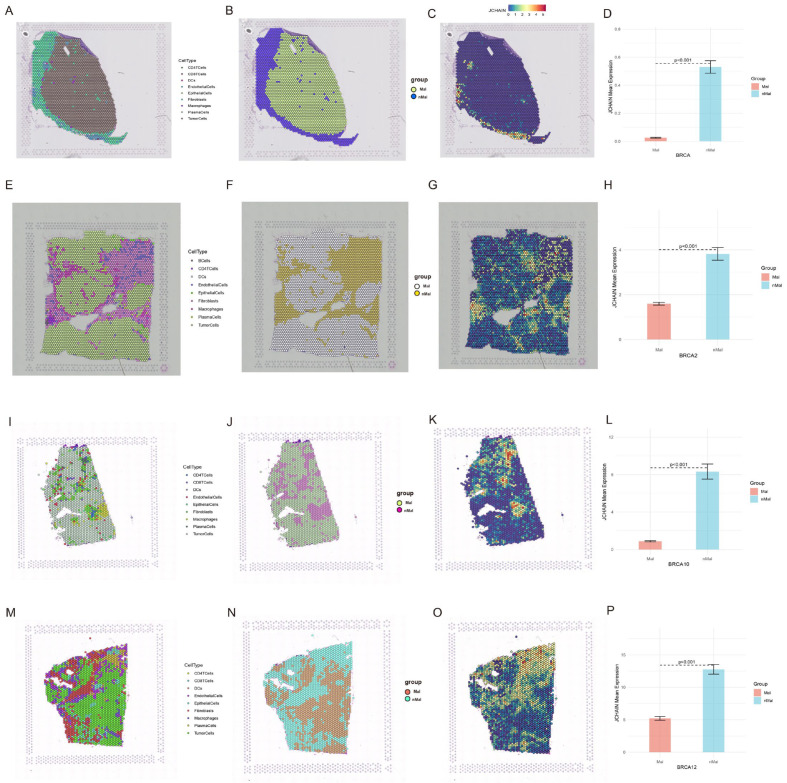
Spatial transcriptomics of *JCHAIN* in BRCA. (**A**–**D**) Distribution of different cells in the GSE203612 dataset (**A**), distribution of malignant (Mal) and non-malignant (nMal) cells (**B**), distribution of *JCHAIN* (**C**), and expression of *JCHAIN* in malignant (Mal) and non-malignant (nMal) cells (**D**). (**E**–**H**) Distribution of different cells in the BRCA_BlockASection1 dataset (**E**), distribution of malignant (Mal) and non-malignant (nMal) cells (**F**), distribution of *JCHAIN* (**G**), and expression of *JCHAIN* in malignant (Mal) and non-malignant (nMal) cells (**H**). (**I**–**L**) Distribution of different cells in the GSE210616-GSM6433587 dataset (**I**), distribution of malignant (Mal) and non-malignant (nMal) cells (**J**), distribution of *JCHAIN* (**K**), and expression of *JCHAIN* in malignant (Mal) and non-malignant (nMal) cells (**L**). (**M**–**P**) Distribution of different cells in the GSE210616- GSM6433589 dataset (**M**), distribution of malignant (Mal) and non-malignant (nMal) cells (**N**), distribution of *JCHAIN* (**O**), and expression of *JCHAIN* in malignant (Mal) and non-malignant (nMal) cells (**P**). Wilcoxon Rank Sum Tests.

The *JCHAIN* expression localisation was determined to be essentially the same as that of B cells and plasma cells using the Sparkle database, and *JCHAIN* expression was low in malignant tissue groups (Mal) and high in non-malignant tissue groups (nMal) (Figure 11A–O). The foregoing results revealed that low *JCHAIN* expression was associated with malignant tissue content, and that *JCHAIN* may influence the cancer microenvironment via B cells and plasma cells.

### 3.10. The JCHAIN Enrichment Analysis in Breast Cancer

**Figure 12:**
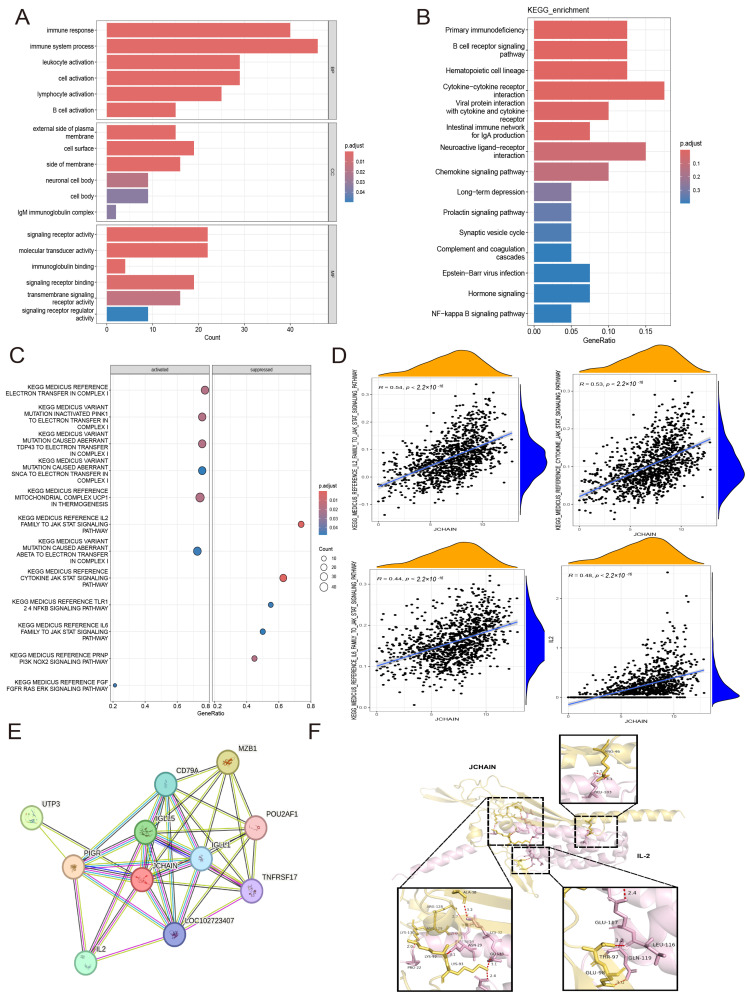
Functional enrichment of *JCHAIN* in BRCA. (**A**,**B**) Functional enrichment of GO and KEGG. (**C**) GSEA pathway enrichment analysis. (**D**) Scatterplots of JAK-STAT and correlation of IL-6 with *JCHAIN*. (**E**) Interaction network of JCHAIN with related proteins and KEGG-enriched pathways in the STRING website. (**F**) Visualisation of JCHAIN and IL-2 protein-protein molecular docking.

After dividing the median expression of *JCHAIN* into high and low expression groups in TCGA and filtering out the differential genes for GO and KEGG enrichment analysis, it was discovered that BP (Biological Process) was primarily enriched in immune response, immune system process, leukocyte activation, cell activation, lymphocyte activation, and B cell activation. The CC (Cellular Component) was primarily enriched in external side of plasma membrane, cell surface, side of membrane. The MF (Molecular Function) was primarily enriched in signalling receptor activity, molecular transducer activity (Figure 12A). KEGG was primarily enriched in signalling pathways, including primary immunodeficiency and the B cell receptor signalling pathway (Figure 12B). These findings match to immune infiltration, single-cell analysis, and spatial histology. Following that, 12 signalling routes related with *JCHAIN* were discovered using GSEA, and correlations were made with these 12 signalling pathways, with the JAK STAT-signalling pathway being identified as the pathway linked with *JCHAIN* (Figure 12C,D and Figure S7A). As a result, the TIMER3 website revealed that *JCHAIN* had the strongest association with JAK3 and STAT4 (Figure S7B,C). After identifying proteins interacting with JCHAIN and KEGG pathway in STRING website (Figure 12E,F), we discovered that JCHAIN can interact with IL2 with a correlation of 0.48 (*p* < 0.05) (Figure 12D). It suggested that JCHAIN may play a key role in breast cancer through the IL2 and JAK-STAT pathways.

### 3.11. The JCHAIN Inhibits Breast Cancer Progression Through IL-2 and STAT4

**Figure 13:**
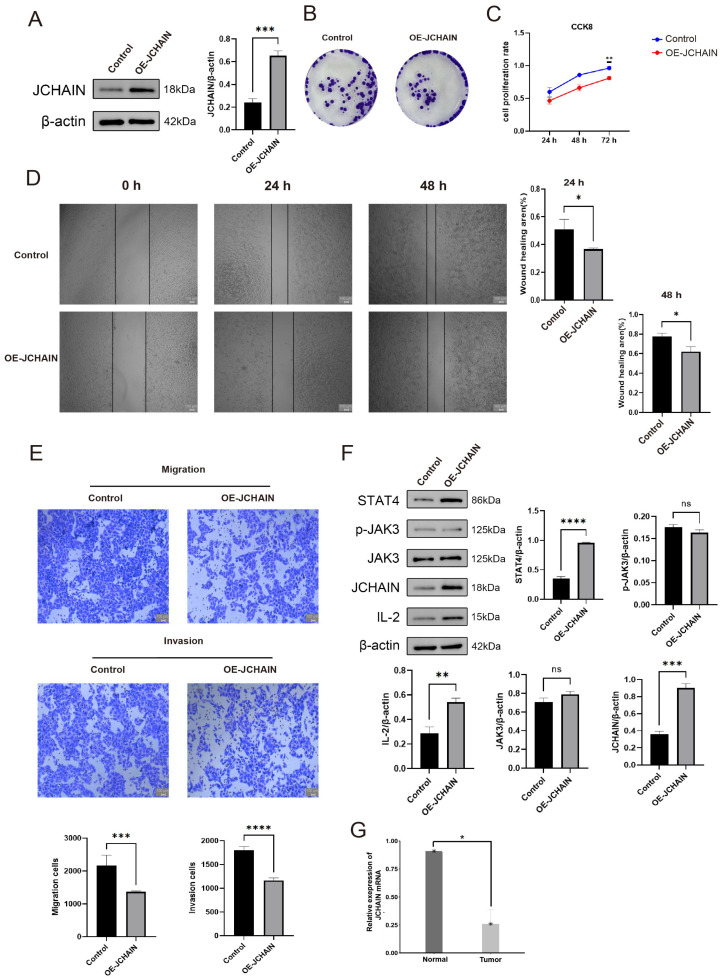
The JCHAIN inhibits breast cancer progression through IL-2 and STAT4. (**A**) JCHAIN protein expression in the Control group as well as in the OE-JCHAIN group. (**B**) Colony generation experiment showed that JCHAIN inhibited breast cancer colony generation. (**C**) The CCK-8 experiment showed that JCHAIN inhibits cell proliferation. (**D**) Cell scratch experiment showed that JCHAIN inhibited breast cancer cell migration. (**E**) Transwell experiment showed that JCHAIN inhibited breast cancer cell migration and invasive ability. (**F**) Immunoblot analysis of STAT4, p-JAK3, JAK3, and IL-2 protein levels after JCHAIN overexpression in breast cancer cells. (**G**) JCHAIN mRNA expression in normal tissues as well as breast cancer tumour tissues. Student’s *t*-test. Ns denotes no statistically significant difference. * *p* < 0.05, ** *p* < 0.01, *** *p* < 0.001, **** *p* < 0.0001.

In this study, we constructed the JCHAIN overexpression plasmid and transfected the control plasmid and the overexpression plasmid into MCF-7 cells, which were divided into the control group (Control) and the JCHAIN overexpression group (OE-JCHAIN). The JCHAIN protein in the OE-JCHAIN group was found to be higher than that in the Control group by WB assay (Figure 13A, *p* < 0.05). The colony generation assay and CCK-8 assay showed that the cell colonisation and proliferation in the OE-JCHAIN group was lower than that in the Control group (Figure 13B,C, *p* < 0.05), and the cell migration rate in the OE-JCHAIN group was lower than that in the Control group in the cell scratch assay at 24 h and 48 h (Figure 13D, *p* < 0.05). Transwell assay revealed that the migration and invasion rates were much higher in the Control group compared with the OE-JCHAIN group (Figure 13E, *p* < 0.05). To further verify how JCHAIN affects cell function, it was verified by protein-protein molecular docking that JCHAIN could bind to IL-2 (Figure 12F), and it was found by protein that the level of IL-2, STAT4 proteins in the OE-JCHAIN group was higher than that in the Control group (Figure 13F). Meanwhile, JCHAIN mRNA was found to be less expressed in tumour tissues than in normal tissues by PCR experiments (Figure 13G). This indicates that JCHAIN may inhibit the proliferation and migration of breast cancer cells by affecting IL-2 and STAT4.

## 4. Discussion

As a key immune modulator, the *JCHAIN* has been extensively studied in various cancers. In a previous study, *JCHAIN* was found to play an important role in human immunity as a chemokine [28]. Streptococcus mongolensis extract was discovered to have an important anti-tumour impact in the treatment of HCC by employing *JCHAIN* as a core target [29]. In breast cancer, the *JCHAIN* inhibits proliferative migration through the NF-KB signalling pathway [6]. We conducted a systematic examination of *JCHAIN*’s role in cancer and discovered that *JCHAIN* gene and protein expression was low in most cancers, including BRCA, COAD, LUSC, HNSC, and LIHC, as measured by pan-cancer analysis, and was also linked with immunostaging and molecular staging. Subsequent prognostic analyses demonstrated that low *JCHAIN* gene expression was related with a poor prognosis in most malignancies, and *JCHAIN* expression was found to be an independent prognostic factor for most cancers using univariate Cox regression. These data show that *JCHAIN* could be employed as a diagnostic and prognostic marker for a wide range of malignancies.

The results of the mutation analysis showed that *JCHAIN* had amplification, mutation, and deep deletion in a variety of cancers, with amplification accounting for the majority. And the unmutated group had a better prognosis than the *JCHAIN* mutated group. This suggests that gene mutations may affect the tumour’s aggressiveness. In a recent study, it was discovered that in *JCHAIN*-deficient mice, steady-state IgM levels were reduced while IgA levels increased, disrupting their relationship [30]. In addition, the *JCHAIN* was found to be negatively associated with aneuploidy, homologous recombination defects (HRDs), mutation rate, and tumour neoantigens (SNV neoantigens) in most cancers, as well as the 30 homologous recombination repair (HRR) signature genes, This shows that DNA damage affects the physiological function of *JCHAIN*.

Methylation is one of the most common epigenetic mechanisms that regulate gene expression, and numerous studies have shown that hypermethylation of tumour-suppressor genes and/or hypomethylation of oncogenes relative to non-tumour tissues is a common feature of many cancers [31]. In this study, we analysed the DNA methylation status on the promoter of *JCHAIN* and found that *JCHAIN* promoter methylation was significantly different in most cancers. In addition, aberrant RNA methylation is closely associated with the development and progression of many cancers [32]. For example, in breast cancer, *ALYREF* promotes breast cancer development as well as affects cell stemness by influencing transcriptional regulation and mitochondrial energy metabolism [33,34], In another study, *YTHDF2* reverses RNA demethylase-induced alterations in cellular phenotype by increasing mRNA stability and promotes cellular proliferation, invasion, and tumorigenic properties in vitro [35]. Therefore, we studied the relationship between RNA methylation signature genes (including m1A, m5C, and m6A) and *JCHAIN* gene expression and found that *JCHAIN* was significantly correlated with RNA methylation signature genes in various cancers. These findings provide new clues for elucidating the epigenetic regulatory mechanisms of *JCHAIN* in tumourigenesis and development.

The *JCHAIN* plays an important role in tumour immunity as an immune-related gene [8,36]. In this study, we examined the correlation between *JCHAIN* and immune infiltration using the TIMER3 website, the Sparkle database, and the TISIDB website. The ESTIMATE algorithm discovered that *JCHAIN* was positively correlated with stroma, immune, and estimate scores, but negatively correlated with tumour scores. *JCHAIN* was also found to be positively correlated with chemokines, chemokine receptors, immunoinhibitors, immunostimulators, MHC signature genes, and lymphocytes (e.g., CD8+ T-cells, B cells, macrophages, and Treg-cells). This was followed by an in-depth study of *JCHAIN* expression in various cancer immune subtypes. The findings revealed significant differences among immune subtypes, which were consistent with previous research [10,37]. Concurrently, flow cytometry analysis revealed that *JCHAIN* expression levels in plasma cells were significantly higher than in B cells. Furthermore, following TRK-fused gene (TFG) knockdown in B cells, *JCHAIN* expression increased, suggesting that *JCHAIN* may participate in the process whereby TFG influences B cell differentiation [38]. The transcription factors interferon regulatory factor 4 (IRF4) and B lymphocyte-induced maturation protein 1 (BLIMP1) play crucial roles in plasma cell differentiation. The present study revealed that *JCHAIN* expression precedes that of *IRF4* and *BLIMP1* during plasma cell differentiation. Furthermore, as plasma cells differentiate, its expression exhibits a strong correlation with IRF4 and BLIMP1 expression [39]. In endometrial carcinoma, a positive correlation between CXCL9 and *JCHAIN* was observed, with *JCHAIN* promoting cytotoxic function [40]. Concurrently, in intrahepatic cholangiocarcinoma (ICC), plasma cells characterised by IGHG1 and *JCHAIN*, alongside immune cell infiltration, portend improved survival rates [41]. These findings collectively suggest that *JCHAIN* may influence the tumour immune microenvironment and patient prognosis by promoting immune cell infiltration and activation.

Our analysis revealed that *JCHAIN* is downregulated in most tumours yet highly expressed in glioblastoma multiforme (GBM) and uveal melanoma (UVM). Reviewing extensive literature on this phenomenon, we found that the tumour microenvironment (TME) of UVM exhibits immunologically ‘cold’ characteristics, manifested as low tumour mutational burden, limited T-cell infiltration, and the presence of numerous immunosuppressive cells [42,43]. Glioblastoma, due to the presence of the blood–brain barrier, similarly possesses a highly suppressive tumour microenvironment, with B cells and plasma cells typically scarce within tumour tissue [44,45]. Within this environment, regulatory B cells influence the function of other immune cells and produce immunosuppressive cytokines—factors that negatively impact prognosis [45,46]. Concurrently, the glioblastoma microenvironment can reshape B cell phenotypes towards immunosuppression and block B cell maturation [47]. We therefore hypothesise that in both tumours, these suppressive B cells may fail to produce antibodies such as IgA and IgM normally, leaving the functional linker *JCHAIN* in an unconsumed state. Consequently, high *JCHAIN* expression in UVMs and GBMs may correlate with poorer prognosis. In contrast, within the microenvironments of other tumours, B cells or plasma cells undergo extensive activation. Here, *JCHAIN* functions as a connecting chain within dimers or pentamers to generate mature antibodies and exert immune functions. Consequently, *JCHAIN* expression is reduced in these tumours. As demonstrated by our study results, *JCHAIN* expression in UVM and GBM shows no significant correlation with B cells, exhibiting even a negative correlation.

The JCHAIN showed significant differences in gene expression, protein expression, prognosis, and immune infiltration in BRCA. The *JCHAIN* protein was found to have a strong association with signalling pathways including as WNT, mTOR, p53, RTK, Hippo, and chromatin remodelling in BRCA. A study discovered that the *JCHAIN* may bind to six genes in ductal breast cancer and could be used as a predictive biomarker for the tumour microenvironment [48]. Another study found that the *JCHAIN* expression in the peripheral blood of cancer patients was substantially linked with prognosis [49]. In this study, low *JCHAIN* expression was related with advanced staging, greater T/N stage, and a poor prognosis by analysis, whereas high expression indicated improved survival. The ROC curve study verified the diagnostic value of *JCHAIN* (AUC = 0.783). Functional enrichment study revealed that *JCHAIN* is mostly involved in B cell activation, immunological response, and the JAK-STAT signalling pathway. Among these, *JCHAIN* had a favourable link with the JAK-STAT signalling pathway and a strong correlation with *JAK3* and *STAT4*. The current investigation discovered that elevated STAT4 expression is associated with a favourable prognosis in breast cancer [50]. In vivo animal investigations revealed that *STAT4* overexpression reversed breast cancer cells’ resistance to radiation and decreased tumour development via increasing THRB expression [51]. *STAT4* expression was also stimulated with an increase in cytotoxic CD4+ T cell numbers, boosting its anti-breast cancer action [52]. This study found that *JCHAIN* can inhibit cell colony formation, migration, and invasion by overexpressing *JCHAIN* in MCF-7 cells. Protein-protein molecular docking revealed that *JCHAIN* binds to IL-2 through 11 hydrogen bonds. WB detection subsequently found that the protein expression of IL-2 and STAT4 in the *JCHAIN* overexpression group was significantly higher than that in the control group. Consistent with previous studies, for example, IL-2 can regulate STAT4 to enhance the function of natural killer cells [53], and it can lead to the down-regulation of STAT4 protein during the treatment of lymphoma, which results in STAT4 defects and affects the therapeutic effect [54].

The link between *JCHAIN* and immune cells in BRCA was then investigated using three algorithms (CIBERSORT, ssGSEA, and ESTIMATE), and it was discovered that *JCHAIN* was negatively connected with M0 and M2 macrophages but favourably correlated with M1. The current study discovered that M1 macrophages increase inflammatory and anti-tumour activities [55,56], but M2 macrophages boost angiogenesis, immune evasion, cancer stemness, tumour cell invasion, and metastasis [57,58]. This also implies that this *JCHAIN* could be linked to macrophage differentiation. Single-cell investigations have revealed that *JCHAIN* is primarily found in plasma cells and B cells in breast cancer. B cells are a key component of adaptive immunity because they produce antibodies, cause cytotoxicity, phagocytose cancer cells, and present antigens [59]. In the current study, tumour-infiltrating B cells have a good prognostic value in solid tumours [60], and B cells can suppress tumours by secreting antibodies and activating T-cell immunity [61]. Another study found that stimulating B cells with CpG-containing oligodeoxynucleotides increased the expression of the TRAIL/Apo-2 ligand, which mediates tumour cell killing [62]. In this study, *JCHAIN* was discovered to be positively linked with B cells in most malignancies, implying that *JCHAIN* modulates the tumour microenvironment and prevents cancer progression via B cells. Furthermore, the spatial transcriptome revealed that *JCHAIN* was strongly expressed in non-malignant spots. These results all suggest that *JCHAIN* may be involved in regulating the immune microenvironment in breast cancer.

In a study conducted on H22 hepatocellular carcinoma-bearing mice, it was discovered that the ethyl acetate extract of Streptococcus mongolicus exerts antitumour effects by enhancing *JCHAIN* protein expression. Notably, Felibomin C, Myosin A, and Feligdin D demonstrated strong binding affinity with *JCHAIN*, indicating its potential as a therapeutic target [29]. immunotherapy for multiple myeloma, *JCHAIN* emerges as a novel target antigen. CD8+ T cells transduced with *JCHAIN*-specific T cell receptors (TCRs) demonstrated potent in vitro and in vivo killing activity against multiple myeloma, confirming *JCHAIN*’s potential as an immunotherapeutic target [36]. Our study further indicates that *JCHAIN* expression levels correlate significantly with patient survival. Elevated expression in most tumours predicts improved prognosis, suggesting *JCHAIN* possesses potential as a novel prognostic biomarker. Furthermore, as a secreted protein present in blood, it facilitates detection [7]. Based on the above evidence, we recommend further validation of *JCHAIN*’s prognostic value in larger, multicentre retrospective cohorts. Concurrently, in-depth study into its specific mechanisms regulating tumour biological behaviour should be pursued. Additionally, active exploration of *JCHAIN*-targeted therapeutic strategies—including small-molecule drug development and innovative immunotherapy approaches—is warranted to advance its clinical translation.

Although we analysed the prognosis as well as the function of the *JCHAIN* in pan-cancer, we must be aware of some limitations. The high correlation of the *JCHAIN* with most immune cells and immune-related genes found in our bioconfidence analyses suggests a potential role for *JCHAIN* in the immune microenvironment, but experiments are needed to further validate this idea. Although we performed in vitro cellular experiments, we lacked in vivo experiments for full validation, as well as validation within multiple cancer cell lines. Therefore, more experiments are needed to further validate the effects of *JCHAIN* on multiple cancers in subsequent studies.

## 5. Conclusions

This study found that *JCHAIN* is a potential diagnostic and prognostic marker in various cancers and is closely related to methylation, cell stemness, and the immune microenvironment in breast cancer. At the same time, cell experiments found that *JCHAIN* may inhibit breast cancer cell proliferation, migration, and invasion through IL-2/STAT4.

## Data Availability

The dataset provided in this study can be downloaded in the online website. TCGA: https://portal.gdc.cancer.gov/ (accessed on 10 June 2024). GEO: https://www.ncbi.nlm.nih.gov/geo/ (accessed on 16 June 2024).

## Supplementary Materials

The following supporting information can be downloaded at: https://www.mdpi.com/article/10.3390/genes16091070/s1, Figure S1: One-way Cox analysis of JCHAIN in pan-cancer; Figure S2: Degree of promoter methylation of JCHAIN in pan-cancer; Figure S3: JCHAIN correlates with RNA methylation modification signature genes in pan-cancer; Figure S4: Relationship between JCHAIN proteins and immune-related pathways; Figure S5: Gene expression of JCHAIN in GEO datasets. Figure S6: Validation of JCHAIN diagnosis and prognosis in the GEO dataset; Figure S7: Scatter plot of the correlation between JCHAIN and GSEA enrichment pathway. Table S1: 30 homologous recombination repair (HRR)-related genes; Table S2: 44 RNA modification genes related to m1A, m5C, and m6A.

## Abbreviations

The following abbreviations are used in this manuscript:

ACC	Adrenocortical Carcinoma
BLCA	Bladder Urothelial Carcinoma
BRCA	Breast Invasive Carcinoma
CESC	Cervical Squamous Cell Carcinoma and Endocervical Adenocarcinoma
CHOL	Cholangiocarcinoma
COAD	Colon Adenocarcinoma
DLBC	Diffuse Large B cell Lymphoma
ESCA	Esophageal Carcinoma
GBM	Glioblastoma Multiforme
HNSC	Head and Neck Squamous Cell Carcinoma
KICH	Kidney Chromophobe
KIRC	Kidney Renal Clear Cell Carcinoma
KIRP	Kidney Renal Papillary Cell Carcinoma
LAML	Acute Myeloid Leukaemia
LGG	Brain Lower Grade Glioma
LIHC	Liver Hepatocellular Carcinoma
LUAD	Lung Adenocarcinoma
LUSC	Lung Squamous Cell Carcinoma
MESO	Mesothelioma
OV	Ovarian Serous Cystadenocarcinoma
PAAD	Pancreatic Adenocarcinoma
PCPG	Pheochromocytoma and Paraganglioma
PRAD	Prostate Adenocarcinoma
READ	Rectum Adenocarcinoma
SARC	Sarcoma
SKCM	Skin Cutaneous Melanoma
STAD	Stomach Adenocarcinoma
TGCT	Testicular Germ Cell Tumours
THCA	Thyroid Carcinoma
THYM	Thymoma
UCEC	Uterine Corpus Endometrial Carcinoma
UCS	Uterine Carcinosarcoma
UVM	Uveal Melanoma
KEGG	Kyoto Encyclopedia of Genes and Genomes
GSEA	Gene Set Enrichment Analysis
TISIDB	Tumor Immune System Interaction Database
