# Supplementary material for: JCHAIN: A Prognostic Marker Based on Pan-Cancer Analysis to Inhibit Breast Cancer Progression
Source: Genes (Basel). 2025 Sep 11;16(9):1070. doi: 10.3390/genes16091070 (PMC12469470; doi:10.3390/genes16091070)
Supplement: Supplementary file 1 [file genes-16-01070-s001.zip › genes-3846847-supplementary.pdf]

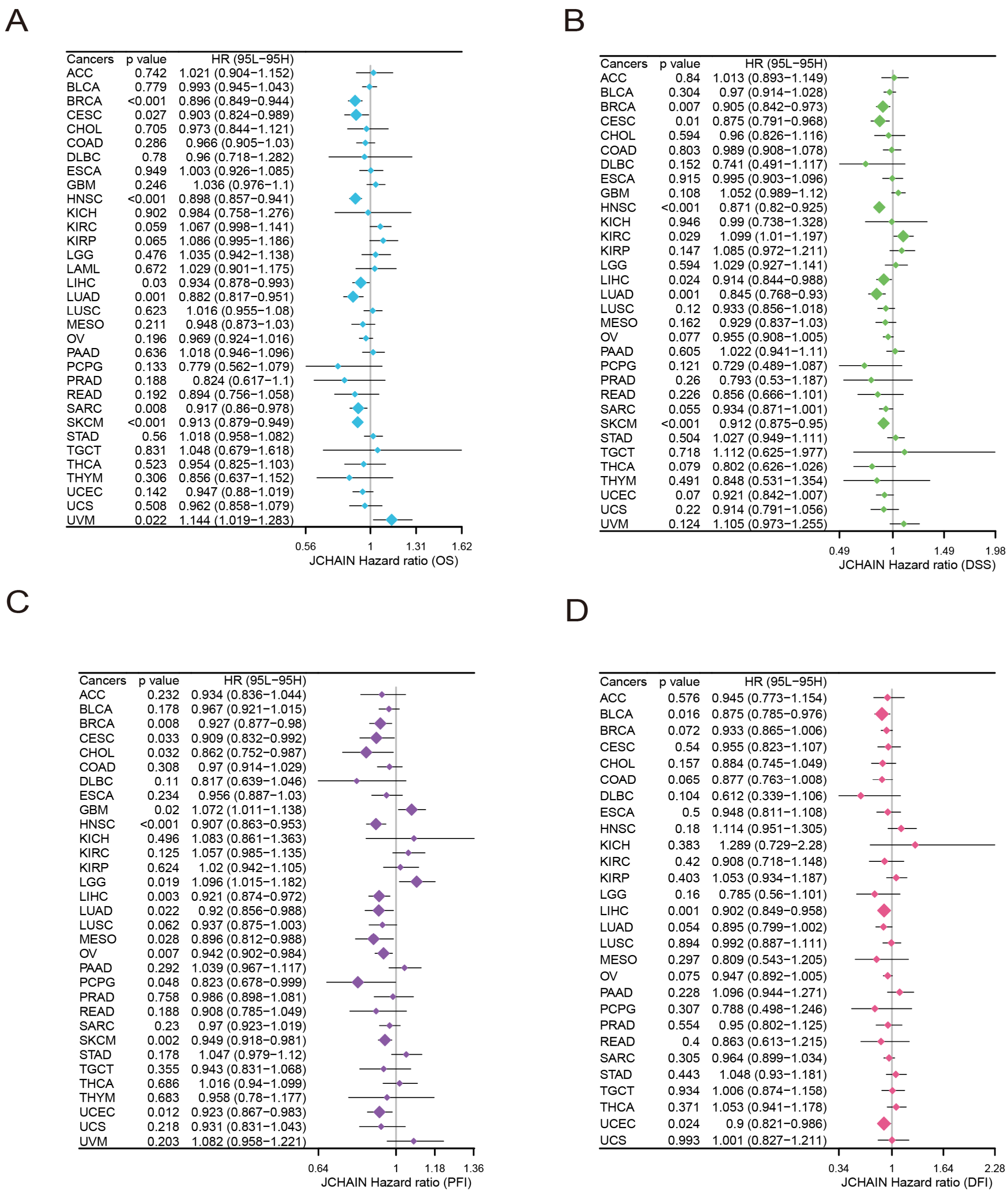

Figure S1. One-way Cox analysis of *JCHAIN* in pan-cancer. (**A**) A one-way Cox analysis was conducted to examine the relationship between the expression of *JCHAIN* and overall survival across various cancer types. (**B**) A one-way Cox analysis was conducted to examine the relationship between the expression of *JCHAIN* and disease specific survival across various cancer types. (**C**) A one-way Cox analysis was conducted to examine the relationship between the expression of *JCHAIN* and progression free interval across various cancer types. (**D**) A one-way Cox analysis was conducted to examine the relationship between the expression of *JCHAIN* and disease free interval across various cancer types.

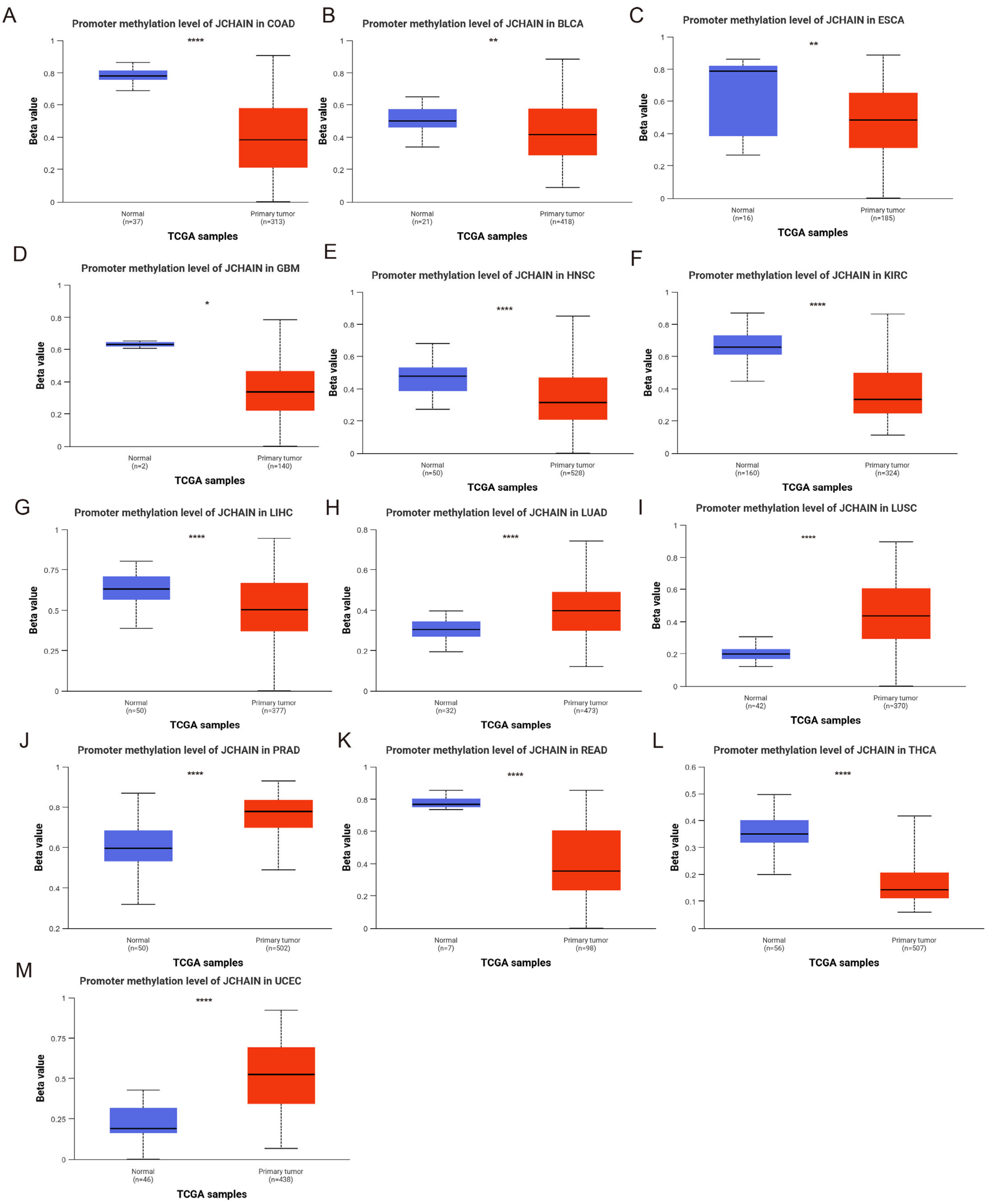

Figure S2. Degree of promoter methylation of *JCHAIN* in pan-cancer. (**A-M**) In the UALCAN tool, *JCHAIN* promoter methylation differs between normal and malignant tissues in COAD (**A**), CESC (**B**), ESCA (**C**), GBM (**D**), HNSC (**E**), KIRC (**F**), LIHC (**G**), LUAD (**H**), LUSC(**I**), PRAD (**J**), READ (**K**), THCA (**L**), and UCEC (**M**). Student's t-test. *P < 0.05, **P < 0.01, ****P < 0.0001.

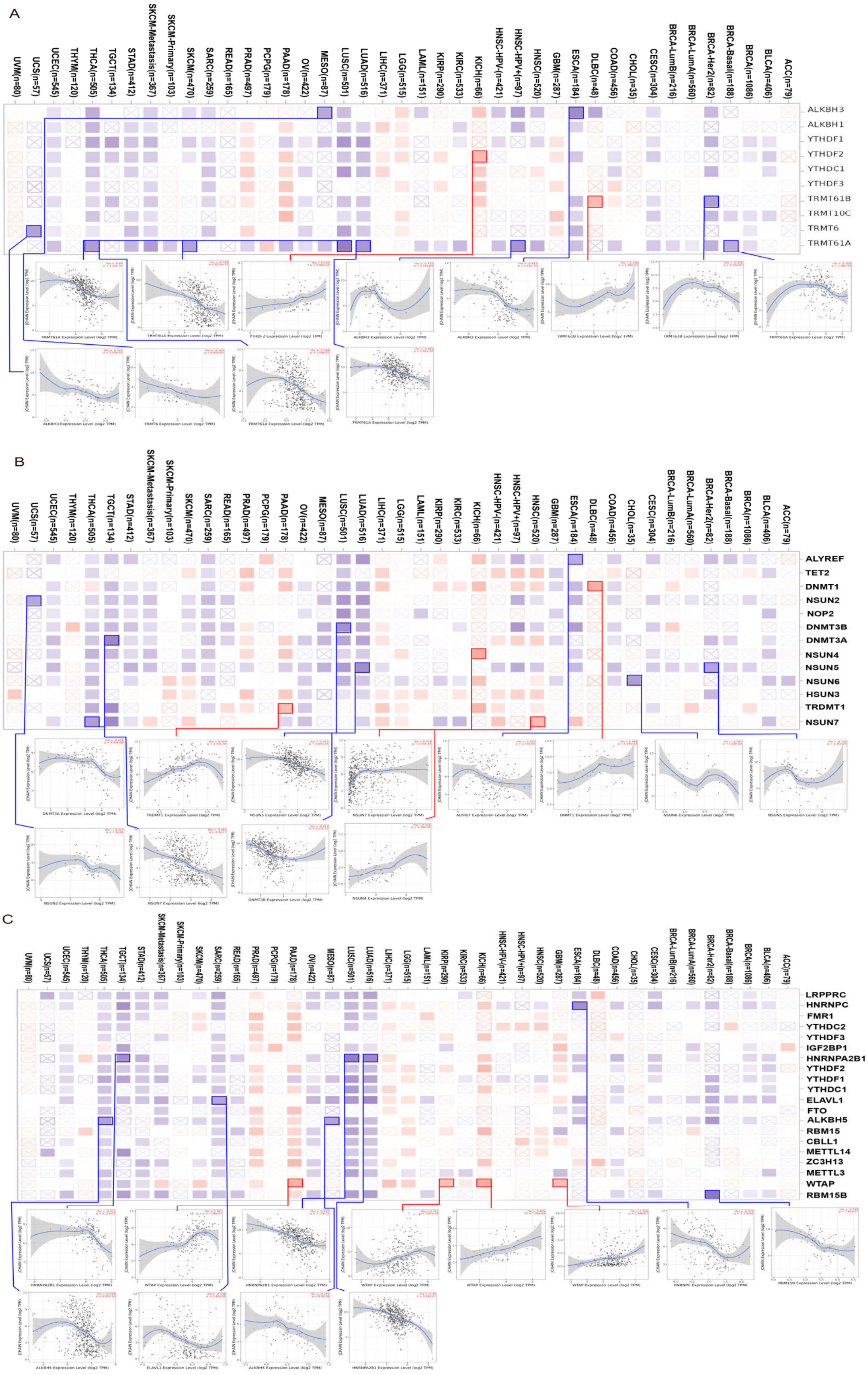

Figure S3. *JCHAIN* correlates with RNA methylation modification signature genes in pan-cancer. (**A-C**) Heatmaps of *JCHAIN* correlation with N6-methyladenosine (m1A), 5-methylcytosine (m5C), and N1-methyladenosine (m6A) signature genes were analysed in TIMER 2.0. Spearman correlation analysis. Blue denotes a negative correlation, while red indicates a positive correlation. The deeper the red or blue hue signifies a correlation approaching 1. A cross symbolises a p-value exceeding 0.05, indicating no significant correlation.

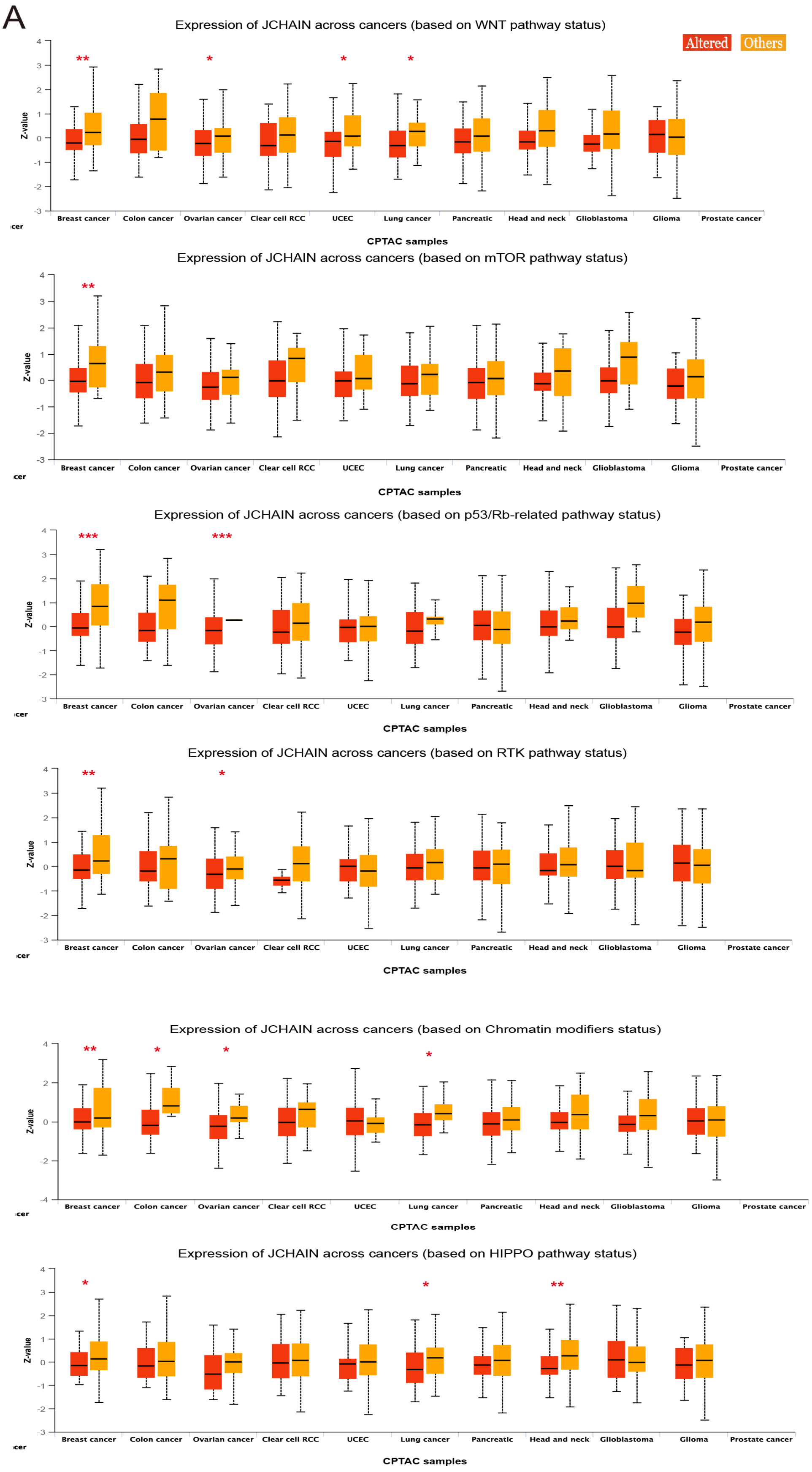

Figure S4. Relationship between JCHAIN proteins and immune-related pathways. (**A**) Box plots of JCHAIN versus WNT, mTOR, p53/Rb-related, RTK, Chromatin modifiers status, and HIPPO pathway expression in the UALCAN tool. Student's t-test. *P < 0.05, **P < 0.01, ***P < 0.001.

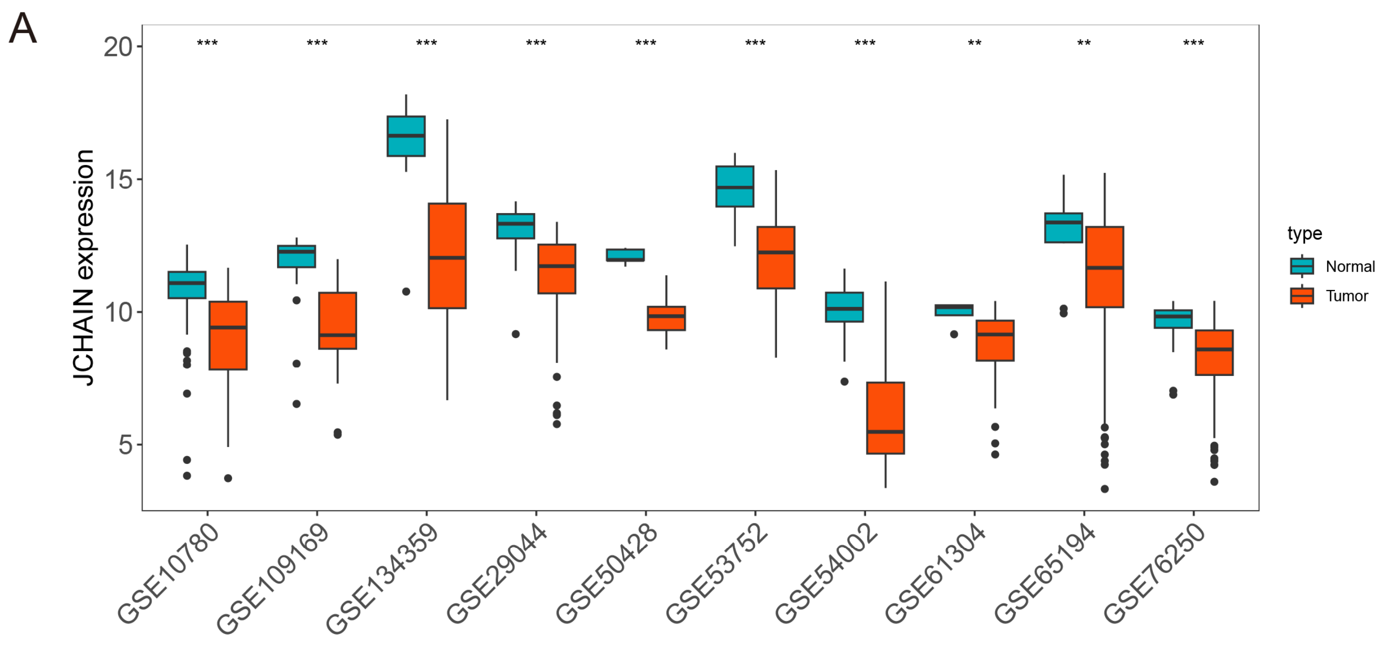

Figure S5. Gene expression of *JCHAIN* in GEO datasets. (**A**) *JCHAIN* expression in normal and tumour samples in GSE10780, GSE109169, GSE134359, GSE29044, GSE50428, GSE53752, GSE54002, GSE61304, GSE65194, and GSE76250 datasets. Wilcoxon Rank Sum Tests. *P < 0.05, **P < 0.01, ***P < 0.001.

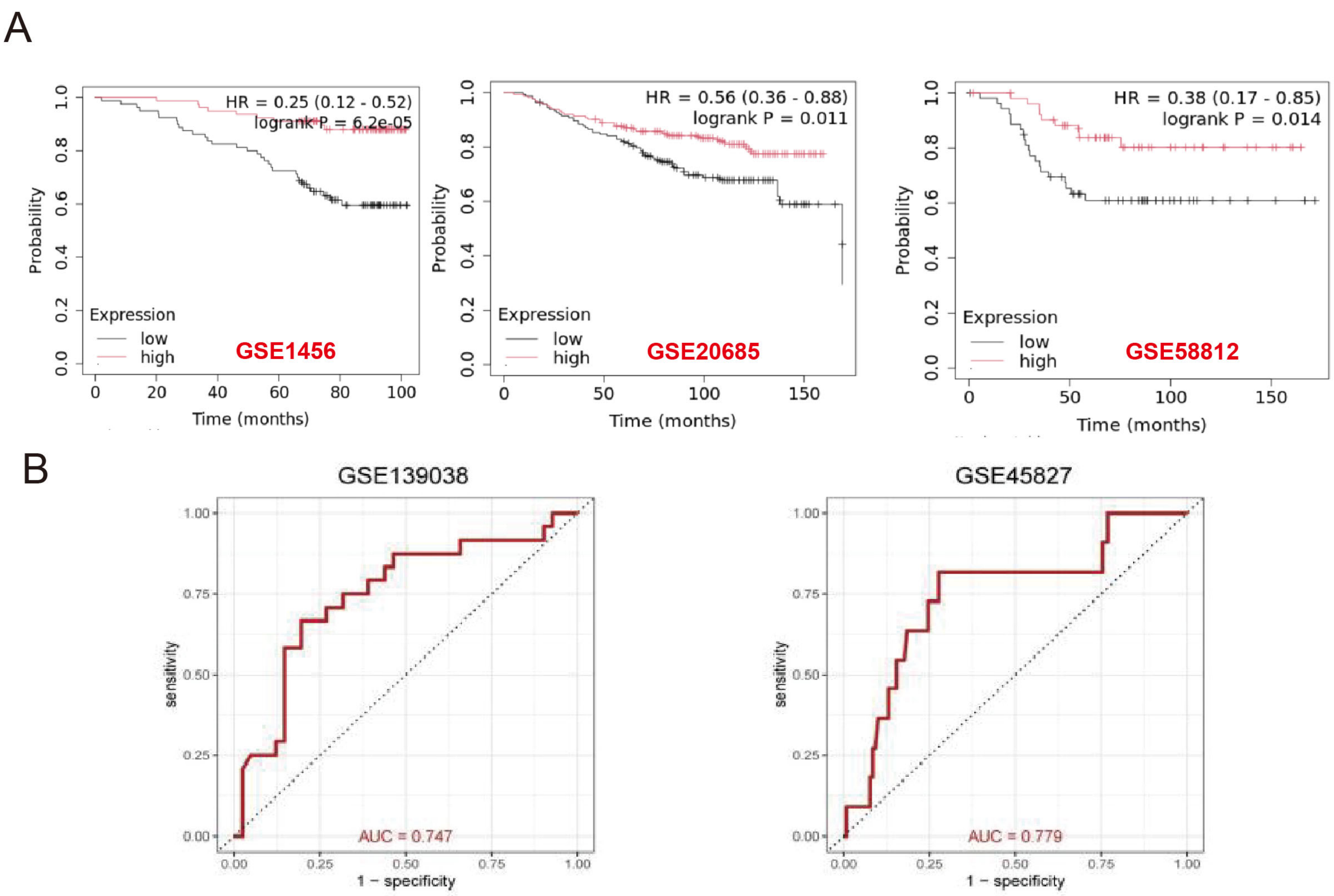

Figure S6. Validation of *JCHAIN* diagnosis and prognosis in the GEO dataset. (**A**) Prognosis of *JCHAIN* was validated in the GSE1456 (left), GSE20685 (Centre), and GSE58812 (Right) datasets via the Kaplan-Meier Plotter website. (**B**) *JCHAIN* ROC curves in the GSE139038 as well as GSE45827 datasets.

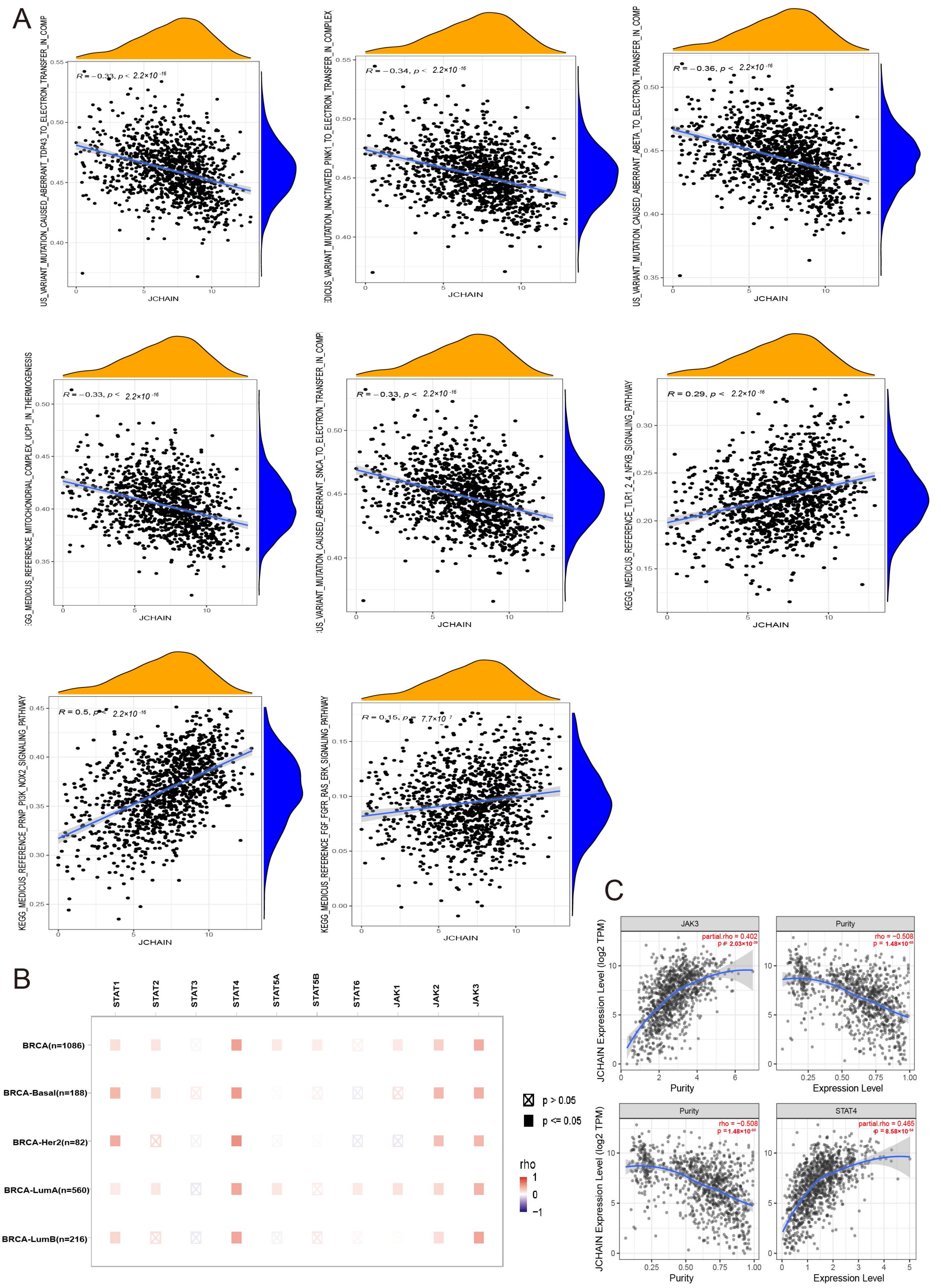
A
R = −0.33, p < 2.2×10 -16
R = −0.34, p < 2.2×10 -16
R = −0.36, p < 2.2×10 -16
R = −0.33, p < 2.2×10 -16
R = −0.33, p < 2.2×10 -16
R = 0.29, p < 2.2×10 -16
R = 0.5, p < 2.2×10 -16
R = 0.15, p = 7.7×10 -7
JCHAIN
KEGG_MEDICUS_REFERENCE_TLR1_2_4_NFKB_SIGNALING_PATHWAY
KEGG_MEDICUS_REFERENCE_PRNP_PI3K_NOX2_SIGNALING_PATHWAY
KEGG_MEDICUS_REFERENCE_FGF_FGFR_RAS_ERK_SIGNALING_PATHWAY
B
STAT1
STAT2
STAT3
STAT4
STAT5A
STAT5B
STAT6
JAK1
JAK2
JAK3
BRCA(n=1086)
BRCA-Basal(n=188)
BRCA-Her2(n=82)
BRCA-LumA(n=560)
BRCA-LumB(n=216)
p > 0.05
p <= 0.05
rho
C
JAK3
Purity
STAT4
partial.rho = 0.402
rho = −0.508
partial.rho = 0.465
JCHAIN Expression Level (log2 TPM)
Expression Level

Figure S7. Scatter plot of the correlation between *JCHAIN* and GSEA enrichment pathway. (**A**) Correlation plot with GSEA enrichment pathway. Pearson correlation analysis. (**B**) Heatmap of the correlation between *JCHAIN* and JAK-STAT related genes. The deeper the red or blue hue signifies a correlation approaching 1. A cross symbolises a p-value exceeding 0.05, indicating no significant correlation. (**C**) Scatterplot of the correlation of *JCHAIN* with JAK3 and STST4.

Table S1. 30 homologous recombination repair (HRR)-related genes.

gene	gene	gene
BRCA1	FANCA	NBN
BRCA2	FANCC	PALB2
ATM	FANCD2	RAD50
ATR	FANCE	RAD51
ATRX	FANCF	RAD51B
BARD1	FANCG	RAD51C
BLM	FANCI	RAD51D
BRIP1	FANCL	RAD52
CHEK1	FANCM	RAD54L
CHEK2	MRE11A	RPA1

Table S2. 44 RNA modification genes related to m1A, m5C, and m6A.

m1A	m5C	m6A
ALKBH3	ALYREF	LRPPRC
ALKBH1	TET2	HNRNPC
YTHDF2	DNMT1	FMR1
YTHDF1	NSUN2	YTHDC2
YTHDC1	NOP2	YTHDF3
YTHDF3	DNMT3B	IGF2BP1
TRMT61B	DNMT3A	HNRNPA2B1
TRMT10C	NSUN4	YTHDF2
TRMT6	NSUN5	YTHDF1
TRMT61A	NSUN6	YTHDC1
	NSUN3	ELAVL1
	TRDMT1	FTO
	NSUN7	ALKBH5
		KIAA1429
		RBM15
		CBLL1
		METTL14
		ZC3H13
		METTL3
		WTAP
		RBM15B
